# Taming the “death receptor”: translating the first-in-class p75^NTR^ modulator LM11A-31 from basic biology, across broad preclinical models, to clinical proof-of-concept

**DOI:** 10.1186/s12967-026-08276-x

**Published:** 2026-05-28

**Authors:** Vanessa F. Langness, Danielle A. Simmons, Sukhneet Kaur, Stephen M. Massa, Frank M. Longo

**Affiliations:** 1 Department of Neurology and Neurological Sciences, Stanford University School of Medicine, 453 Quarry Road, Stanford, CA 94305 USA; 2 Wu Tsai Neuroscience Institute, Stanford University, CA 94305 Stanford, USA; 3 Department of Neurology, San Francisco Veterans Affairs Health Care System, 4150 Clement Street, San Francisco, California 94121 USA; 4 Department of Neurology, University of California, San Francisco, California 94121 USA

**Keywords:** P75^NTR^, LM11A-31, Neurotrophins, Neurodegeneration, Dendritic spines, Synapse, Inflammation, Alzheimer’s disease, Huntington’s disease, HIV, Injury

## Abstract

**Background:**

The p75 neurotrophin receptor (p75^NTR^) is a critical regulator of diverse biological processes. Depending on the cellular context, p75^NTR^ can promote trophic or degenerative signaling, which can influence a broad spectrum of pathological conditions, including neurodegenerative diseases, inflammatory/infectious conditions, and various central and peripheral nervous system injuries. These attributes of p75^NTR^ and its widespread, frequently upregulated expression on affected cells and tissues, make it a compelling therapeutic target. Among various therapeutic targeting strategies, the first-in-class small molecule p75^NTR^ modulator, LM11A-31, has emerged as a leading candidate and has been evaluated in 62 published preclinical studies spanning 26 distinct disease and injury models.

**Main body:**

This review covers the foundational biology of p75^NTR^ signaling and expression and all published mechanistic and preclinical studies evaluating LM11A-31, focusing on outcomes that have been reproduced across multiple studies and independent labs. Beyond its therapeutic potential, we also explore how LM11A-31 has served as a powerful pharmacological probe, significantly advancing our knowledge of p75^NTR^ biology. LM11A-31 consistently reduced elevated JNK/c-Jun, NFκB, and RhoA signaling, and normalized reduced PI3K/AKT signaling in a variety of pathological conditions. It also mitigated associated disease processes, including inflammation, degeneration of neurites, synapses, and dendritic spines, and cell death. These protective effects against pathologies often improved functional outcomes, including cognitive and motor measures. Studies using LM11A-31 also uncovered emerging molecular and cellular functions of p75^NTR^ by demonstrating its ability to improve autophagy, calcium and redox homeostasis, and blood-brain barrier function. Finally, a Phase 2a clinical trial applying LM11A-31 for Alzheimer’s disease demonstrated a favorable safety profile and significantly slowed the progression of biomarkers related to neurodegenerative mechanisms.

**Conclusions:**

Together, these studies expand our understanding of p75^NTR^ function and support p75^NTR^ modulation as a promising therapeutic strategy across diverse pathological conditions.

## Introduction

The p75 neurotrophin receptor (p75^NTR^) has emerged as an integral regulator of a broad range of biological functions, including cell death, survival, metabolism, and tissue repair, since its discovery in 1973 and cloning in 1986 [1–4]. This broad functionality results from the complexity of p75^NTR^ signal transduction, which, depending on its autonomous activity or interactions with a plethora of ligands, co-receptors, and intracellular adaptor proteins, can promote trophic or degenerative signaling, making it relevant to a variety of disease and injurious states. Accordingly, p75^NTR^ effects have been implicated in diverse pathological conditions, including neurodegenerative diseases (e.g., Alzheimer’s (AD), Huntington’s (HD), Parkinson’s (PD), multiple sclerosis (MS), and amyotrophic lateral sclerosis (ALS)), hypoxia-related brain damage, obesity, type 2 diabetes, inflammation- and infection-related conditions as well as traumatic brain, spinal cord, peripheral nerve, and skeletal muscle injuries [4–8]. Moreover, p75^NTR^ transcript and/or protein levels are upregulated in many pathological states. Given these distinctive attributes, p75^NTR^ is an intriguing focus for therapeutic development, providing the possibility of promoting trophic/survival signaling and inhibiting degenerative signaling by modulating a single target. p75^NTR^ has been targeted via genetic manipulation to reduce the receptor’s expression or function and via peptides or small molecules that can normalize p75^NTR^ levels and modulate its signaling [4, 9]. Of these, the most widely studied and applied is the first-in-class p75^NTR^ small-molecule modulator, LM11A-31, which will be the focus of this review. First, p75^NTR^ signaling mechanisms and tissue expression will be reviewed to provide the context for understanding LM11A-31’s mechanistic effects and how small-molecule modulators selectively engage pathways distinct from those triggered by p75^NTR^’s native ligands. We then discuss the discovery, characterization, and validation of LM11A-31, its application across a wide range of preclinical studies, and its advancement to human clinical trials. Beyond LM11A-31’s therapeutic potential, we also explore how its preclinical application has served as a powerful probe to markedly expand our understanding of p75^NTR^ mechanisms and the scope of its effects in health and disease.

### p75^NTR^ signaling mechanisms

An overview of p75^NTR^ signaling mechanisms provides the context needed to evaluate the modes of action, effects, and potential novel applications of small molecule modulation of this receptor. p75^NTR^ is a 75 kDa single transmembrane protein and a member of the tumor necrosis factor receptor (TNFR) superfamily (TNFR16); it contains 4 cysteine-rich structures in its extracellular domain (ECD), which control receptor configuration and ligand binding [10, 11]. It also contains a transmembrane domain and an intracellular domain (ICD) containing a juxtamembrane protein-binding region and a C-terminal globular death domain. The ICD provides docking sites for intracellular adaptor proteins. p75^NTR^ relies on its interactions with these adaptor proteins, and in some cases, co-receptors, to activate or modulate signal transduction in response to ligand binding since its ICD lacks catalytic activity [4, 11]. The various adaptor and co-receptor combinations enable p75^NTR^ ligands to elicit differential downstream effects [4, 9].

p75^NTR^ serves as a functional receptor for neurotrophins, as well as other, non-neurotrophin ligands, including the amyloid-β (Aβ) peptide of the amyloid precursor protein, rabies virus glycoprotein, and a fragment of the prion protein [4, 5, 12]. Neurotrophins are a family of secreted growth factors that promote the survival, maturation, differentiation, and function of multiple cell types, including neurons and glia [5]. They also influence cell migration, axonal outgrowth, synapse and dendritic spine integrity, synaptic plasticity, inflammation, and response to injury in the central and peripheral nervous systems (CNS, PNS) [5, 13]. The mammalian neurotrophins are nerve growth factor (NGF), brain-derived neurotrophic factor (BDNF), neurotrophin 3 (NT3), and neurotrophin 4/5 (NT4/5) [9, 14]. They are synthesized as proneurotrophin precursor proteins that are cleaved to their mature forms. Each mature neurotrophin binds with high affinity to its cognate tropomyosin receptor kinase (Trk), activating canonical Trk signaling: NGF binds to TrkA; BDNF and NT4/5 to TrkB; and NT3 to TrkC [13]. Mature neurotrophins also bind with low-affinity to p75^NTR^ while their precursor forms, including proNGF and proBDNF, bind with higher-affinity [9, 14]. Proneurotrophins are the dominant ligand for p75^NTR^ in the CNS [15–17].

**Fig. 1:**
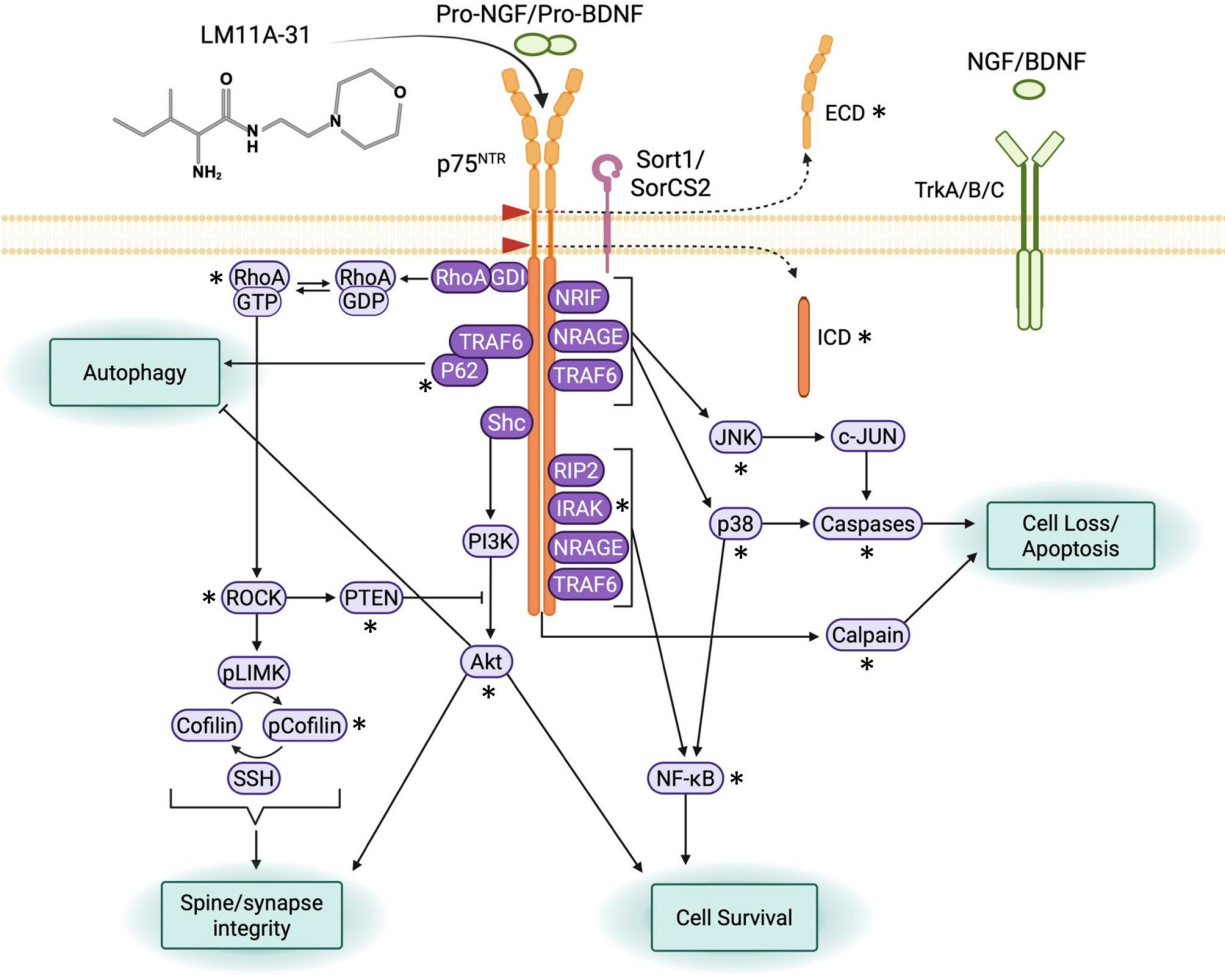
p75^NTR^ signaling in neurons: modulation by LM11A-31. In neurons, p75^NTR^ signaling regulates pathways controlling cell survival and loss/apoptosis, dendritic spine and synapse integrity, and autophagy. p75^NTR^ can couple to diverse intracellular adaptors and signaling proteins to activate either trophic or degenerative signaling cascades. Proneurotrophins bind with high affinity to p75^NTR^ in complex with sortilin 1 (Sort1) or SorCS2 to activate degenerative pathways. Mature neurotrophins bind to p75^NTR^ with low affinity, allowing p75^NTR^ to potentiate Trk-mediated trophic signaling. LM11A-31 promotes trophic signaling and inhibits degenerative signaling. Red arrows indicate the ADAM17/TACE cleavage site on p75^NTR^ that releases the extracellular domain (ECD), and the γ-secretase cleavage site that releases the intracellular domain (ICD). Dark purple ovals indicate adaptor proteins that interact with p75^NTR^, light purple ovals indicate signaling proteins downstream of p75^NTR^, teal boxes indicate a cellular or physiological response. Intracellular signaling intermediates shown to be affected by LM11A-31 are indicated by an asterisk (*)

**Fig. 2:**
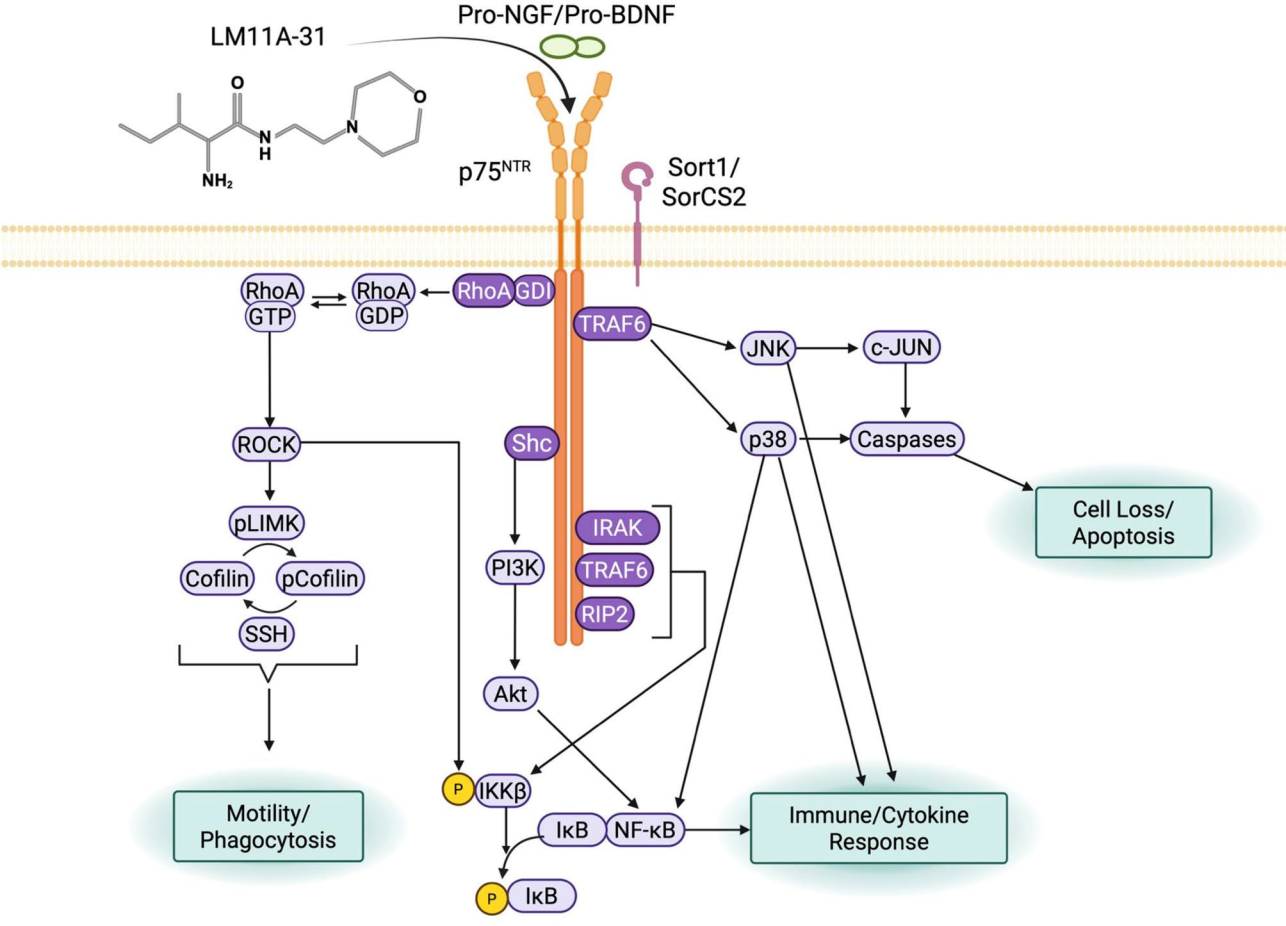
Model of p75^NTR^ signaling in microglia: potential effects of LM11A-31. In microglia, p75^NTR^ couples to diverse intracellular adaptors and signaling proteins to regulate pathways controlling cell survival or apoptosis, motility, phagocytosis, and immune response/cytokine production. Dark purple ovals indicate adaptor proteins that interact with p75^NTR^, light purple ovals indicate signaling proteins downstream of p75^NTR^, teal boxes indicate a cellular or physiological response

p75^NTR^’s interactions with its varied co-receptors and adaptor proteins, initiate diverse downstream signaling pathways that confer divergent functions. Depending on its binding partners, p75^NTR^ can promote trophic or degenerative signaling through several major pathways, including phosphoinositide 3-kinase (PI3K)/Akt, nuclear factor binding near the κ light-chain gene in B cells (NFκB), c-Jun N-terminal kinase (JNK), and Ras homolog family member A (RhoA)/Rho-associated protein kinase (ROCK)/phosphatase and tensin homologue (PTEN) signaling (Figs. 1 and 2) [4]. p75^NTR^ can interact with Trk receptors to enhance their affinity to mature neurotrophins, which activates signaling via mitogen-activated protein kinase (MAPK)/extracellular signal-regulated kinase (ERK), PI3K/Akt, and phospholipase C-gamma (PLCγ)/protein kinase C (PKC) pathways to promote cell survival and dendritic spine/synapse integrity [4, 13]. Mature neurotrophin binding to p75^NTR^ can also initiate interactions between the receptor’s ICD and receptor-interacting protein 2 (RIP2), interleukin-1 receptor–associated kinase (IRAK), neurotrophin receptor-interacting melanoma antigen-encoding gene homologue (NRAGE), or TNF receptor-associated factor 6 (TRAF6), leading to NFκB activation [4, 9, 18–20]. Activating NFκB primarily promotes transcription of pro-survival genes in neurons and pro-inflammatory genes in microglia [21] (Figs. 1 and 2).

Proneurotrophins, like proNGF or proBDNF, can signal through p75^NTR^ when it forms a complex with its co-receptor sortilin, which binds to the neurotrophin’s pro-domain [22, 23]. This complex recruits adaptor proteins, including neurotrophin receptor-interacting factor (NRIF), NRAGE, and TRAF6, which activate MEK kinase (MEKK) [9, 24–26] (Figs. 1 and 2). Activated MEKK phosphorylates and activates both JNK and p38, which promotes downstream signaling that decreases expression of anti-apoptotic proteins, increases expression of pro-apoptotic proteins, and promotes caspase activation and apoptosis [13, 14, 26, 27]. JNK and p38 activation also promote microglia, T-cell, and B-cell activation via transcription factors, such as those in the NFκB protein family, which up-regulate pro-inflammatory mediators, including cytokines (e.g. interleukin (IL)-1β and IL-6), and cyclooxygenase-2 (COX-2) [28, 29]. p75^NTR^ may also form complexes with other signaling partners, such as ephrin-As to activate Fyn, a Src-family kinase involved in synaptic plasticity and tau phosphorylation [30]. Fyn phosphorylates tau at Y18 [31] and the N-methyl-D-aspartate receptor (NMDAR) NR2B subunit at Y1472 [32], events that influence tau aggregation [33] and NMDAR stabilization at synapses [34]. Finally, Aβ oligomers can bind to p75^NTR^ to activate the JNK-c-Jun pathway mediating hippocampal neuron death [35, 36].

Unliganded p75^NTR^, or binding of pro-BDNF, can activate signaling by the small GTPase RhoA to induce diverse fundamental cellular effects [4, 37–40]. These effects include reducing autophagy, actin filament remodeling essential for neurite outgrowth, dendritic spine plasticity, and microglial motility. RhoA activation can also promote apoptosis, microglial phagocytosis, and inflammatory phenotypes. Conversely, mature neurotrophin binding to p75^NTR^ suppresses RhoA signaling to oppose these effects. Other ligands that can activate p75^NTR^-mediated RhoA signaling include myelin-derived proteins such as myelin-associated glycoprotein (MAG), the extracellular domain of Nogo-A (Nogo-66), and oligodendrocyte-myelin glycoprotein (OMgp). These ligands bind to the Nogo receptor, which forms a signaling complex with LRR and Ig domain-containing, Nogo receptor-interacting protein (LINGO) and p75^NTR^ to activate RhoA via p75^NTR^ signaling, leading to neurite outgrowth inhibition [41, 42]. The cellular effects of RhoA signaling are mediated by various downstream effectors (Figs. 1, 2). RhoA activates ROCK, which can phosphorylate and activate PTEN, a negative regulator of the pro-survival PI3K/AKT pathway. RhoA signaling can also activate the pro-apoptotic p38 and JNK signaling pathways [40]. ROCK phosphorylates LIM kinase (LIMK), which then phosphorylates and inactivates the actin-depolymerizing protein cofilin, thus inhibiting actin remodeling, enlarging existing neuronal dendritic spines and neurites while preventing structural plasticity required for new growth, causing neurite retraction and reduced dendritic spine density (Fig. 1).

Dysregulation of RhoA signaling in either direction can disrupt spine and synapse integrity. In microglia, RhoA/ROCK-mediated effects on cofilin and actin remodeling also influence migration and phagocytosis (Fig. 2). RhoA/ROCK activation may be necessary to maintain a pro-inflammatory phenotype as ROCK inhibition downregulates microglial phagocytosis [43], promotes an anti-inflammatory phenotype in microglia [44], and RhoA/ROCK signaling promotes microglial release of inflammatory factors such as IL-1β, IL-6, TNF-α, and nitric oxide by activating NFκB [40]. ROCK phosphorylates the IкB kinase subunit β (IKKβ), which phosphorylates the inhibitor of NFкB (IкB), triggering its degradation and the release of NFкB, which can then translocate to the nucleus where it activates transcription of proinflammatory factors [40] (Fig. 2).

p75^NTR^ can also signal via its proteolytic products. Ligand binding to p75^NTR^ can trigger regulated intramembrane proteolysis of the ectodomain by TNF-α converting enzyme (TACE)/a disintegrin and metalloproteinase 17 (ADAM17), generating a free ECD and a membrane-bound CTF (Fig. 1). CTF is further cleaved by γ-secretase, releasing a p75^NTR^-ICD fragment [10, 14, 45] that can translocate to the nucleus to influence expression of hypoxia-induced genes [13], interact with Trk receptors to potentiate trophic signaling [13], or, in some cases, promote pro-apoptotic signaling [9, 46, 47]. Notably, genetic variants in genes related to p75^NTR^ cleavage and signaling have been linked to AD. A loss-of-function mutation in the ADAM17 gene increased risk of late-onset AD [48], and a variant in the sortilin-1 gene is associated with reduced AD risk [49].

### p75^NTR^ levels are upregulated in pathological conditions

During development, p75^NTR^ is widely expressed in both the CNS and PNS, where it is present in multiple brain regions and cell types, including neurons, astrocytes, microglia, monocytes, and macrophages [5, 50, 51]. In the healthy adult CNS, p75^NTR^ levels remain high in the basal forebrain, particularly in cholinergic neurons and sympathetic neurons. Expression of p75^NTR^ is down-regulated but persists at low levels in the cerebellum, septum, striatum, medulla, spinal cord, and hippocampus, where it is also present on dendritic spines [5, 19, 52, 53]. Its expression also persists on other cell types, including Schwann cells, microglia, monocytes, macrophages, and adult neural progenitor cells [5, 51, 53]. Besides the brain and spinal cord, p75^NTR^ is expressed in retina, cochlea, liver, and white adipose tissue [4, 53].

While p75^NTR^ is more heavily expressed during development than in adulthood, its levels are increased in settings of brain injury, disease, inflammation, and aging [5, 12, 19, 54] in various cell types, including neurons [55], astrocytes [56], microglia [57], and oligodendrocytes [58]. p75^NTR^ expression is upregulated in cell types and tissues that are affected by various neuropathological states in humans and/or mouse models, including but not limited to, cortical and basal forebrain cholinergic neurons in AD [5, 59–61], medium spiny neurons and parvalbuminergic interneurons of the striatum in HD [8, 62], dopaminergic neurons of the substantia nigra in PD [46, 63], cortical neurons after traumatic brain injury (TBI) [5, 64], ischemic tissue following stroke [65], motor neurons in ALS [66], motor neurons and Schwann cells with spinal cord injury [67], dorsal nerve bundles after peripheral nerve injury [68], skeletal muscle after sciatic denervation [69], cortex and hippocampus with bacterial meningitis [70], and human cell lines from malignant gliomas [13].

Regarding glial cells, p75^NTR^ is upregulated on oligodendrocytes in MS, HD, and following spinal cord injury, where it has a well-characterized role in oligodendrocyte apoptosis in response to high neurotrophin or proneurotrophin levels [4, 71]. p75^NTR^ expression is upregulated on astrocytes in response to NGF [50] and during bacterial meningitis [70], which may be related to the proliferation of these glia. Finally, p75^NTR^ is integrally involved in inflammatory responses and related signaling [19], and its expression is increased in immune cells and microglia under various pathological conditions, including splenic monocytes with skeletal muscle injury [7], macrophages with viral infection [72], and in microglia of AD and MS patient brains [73, 74].

An imbalance of p75^NTR^ to Trk receptors occurs in affected cells or tissues of multiple human and/or mouse models of pathological conditions, which can increase pro-apoptotic signaling and reduce pro-survival signaling. A p75^NTR^/TrkA imbalance occurs in the basal forebrain in AD [75], synovial tissue and mononuclear cells in juvenile idiopathic arthritis [76, 77], and in the urothelium of the bladder with cystitis [78]. A p75^NTR^/TrkB imbalance has been reported in HD striatum and cortex [8] and in skeletal muscle after injury [69]. In addition to shifts in levels of p75^NTR^ and Trks, increased levels of precursor neurotrophin forms occur in pathological states, creating a proneurotropin/neurotrophin imbalance that can affect p75^NTR^ signaling. For instance, in aging and AD, there is a higher proNGF/NGF ratio, which has been associated with neurodegeneration [16, 75, 79]. In early stages of AD, including mild cognitive impairment, proNGF levels in CSF are significantly increased and have been proposed as an early-stage biomarker for AD [80]. proNGF/NGF increases are also seen in diabetic retinopathy [81] and spinal cord injury [58, 67]. Elevated proBDNF is reported in skeletal muscle following injury [69], brain tissue with ischemic stroke [82], and with sepsis [83]. Finally, NGF/p75^NTR^ is down-regulated in fibroblasts from patients with Rett Syndrome [84].

The broad range of diseases with altered p75^NTR^ expression and signaling makes the receptor a versatile therapeutic target. Moreover, settings in which proneurotrophins are elevated are potentially especially amenable to a p75^NTR^ blocking strategy.

### Discovery and characterization of the p75^NTR^ ligand LM11A-31

Recognizing the diversity of functions mediated by p75^NTR^ and its broad therapeutic potential, our laboratories began initial work to develop ligands that modulate p75^NTR^ signaling. We profiled synthetic peptides with sequences that correspond to potential receptor-binding domains within NGF, identifying linear peptides with a Lys-Gly-Lys-Glu (KGKE) sequence that inhibited NGF activity in vitro [85]. KGKE was subsequently shown to comprise the NGF β-hairpin loop 1 domain, which interacts with p75^NTR^ [86]. A cyclized, dimeric form of a KGKE-containing synthetic peptide was shown to promote cell survival in a p75^NTR^-dependent fashion, and was the first synthetic peptide to exhibit p75^NTR^-dependent neurotrophic activity [9, 87]. Another cyclic peptide was later developed that mimics the KGKE-containing p75^NTR^-binding loop of NGF and also functions as a p75^NTR^ antagonist (CATDIKGAEC) [88], further confirming the role of the NGF β-hairpin loop 1 domain in p75^NTR^ interactions. Interestingly, this peptide protected neuronal cultures against Aβ-induced cell death and JNK activation and prevented Aβ-induced cortical inflammation in mice, consistent with the findings that Aβ binds to p75^NTR^ [35, 89].

Pharmacophores modeled from a portion of NGF loop 1 containing the KGKE peptide were used to screen small-molecule libraries for non-peptide, small molecules that mimic key structural and physical-chemical features of NGF loop 1 with the intent that such compounds might engage p75^NTR^ to modulate its activity [90]. This in silico screening identified ~60 candidate ligands, of which 35 were obtainable from commercial sources; 23 of these were water soluble and used for in vitro neuronal assays measuring neuronal survival. Four of them, LM11A-7, −24, −28, and −31, promoted the survival of mouse hippocampal neurons in in vitro assays [90]. These four small molecules were then further profiled for dose-response relationships and p75^NTR^ dependency of their effects on neuronal survival. Both LM11A-24 (a caffeine derivative) and LM11A-31 (an isoleucine derivative) showed potent p75^NTR^-dependent neurotrophic activity and favorable drug-like properties [91]. LM11A-24 was subsequently found to produce therapeutic effects in a cellular model of ALS [92] as well as mouse models of AD [67, 93, 94] and spinal cord injury [95] and remains a viable lead compound. LM11A-31 [2-amino-3-methyl-pentanoic acid (2-morpholin-4-yl-ethyl)-amide; MW of the free base is 243.35; see structure in Figs. 1 and 2] was determined to be somewhat more pharmacologically favorable and was ultimately advanced for further study.

Multiple lines of evidence suggest that LM11A-31 interacts with p75^NTR^ and can modulate signaling through the receptor. LM11A-31 displaced NGF from p75^NTR^ but not TrkA and rapidly recruited the p75^NTR^ signaling adaptor IRAK but did not activate TrkB in hippocampal neurons or TrkA in 3T3-TrkA cells [90]. It also inhibited proNGF binding in 3T3-p75^NTR^ cells in vitro [90] and in spinal cord in vivo [67]. In addition, it did not bind to any of a panel of 55 common receptors, as indicated by a Cerep binding inhibition screen [96]. LM11A-31 failed to elicit Akt signaling or trophic activity in p75^NTR-/-^ hippocampal neurons [90] or proliferation and survival of p75^NTR-/-^ NPCs [64], effects that were present in p75^NTR+/+^ cells. Moreover, the pro-survival effects of LM11A-31 on hippocampal neurons and NPCs were blocked with an antibody targeting the p75^NTR^ binding site [90]. LM11A-31 prevented proneurotrophin-induced cell death in mature oligodendrocytes and at higher concentrations it inhibited proNGF binding to p75^NTR^ [64, 90]. Finally, LM11A-31 inhibited the interaction of proNGF and p75^NTR^ in the spinal cord in a mouse model of spinal cord injury [67]. These multiple orthogonal approaches support a model in which LM11A-31 acts through p75^NTR^, independent of Trks, to promote survival signaling, inhibit degenerative signaling, and block proneurotrophin-mediated death. These findings are consistent with complex modulation of the receptor; however, in settings where proneurotrophins promote degeneration or related effects, and/or where the dominant function of p75^NTR^ is degenerative, LM11A-31 may be described as a p75^NTR^ antagonist.

Subsequently, two small-molecule p75^NTR^ antagonists, Thx-B and EVT901, have been developed by other groups [4]. Thx-B is a derivative of LM11A-24 that has shown beneficial effects across models of retinal degeneration, diabetic neuropathy, and ischemic skeletal muscle injury. EVT901, a piperazine derivative developed by Evotec, reduced neuroinflammation in a rat model of AD and improved outcomes in rat models of TBI. The outcomes achieved with these additional small molecules further support p75^NTR^ as a therapeutic target. Since LM11A-31 is the most extensively characterized p75^NTR^ modulating compound and the only one that has as yet advanced into human testing, it is our focus for further discussion below.

Pharmacokinetic studies have shown that LM11A-31 can cross the blood-brain barrier (BBB) and reach effective CNS concentrations. At a single oral dose of 50 mg/kg, LM11A-31 concentrations peaked at ~ 1076 nM in the C57BL/6 mouse brain after 30 min and demonstrated a brain half-life of ~ 4 hours [93]. This peak level exceeded the amount needed for neuroprotection in vitro (e.g., the EC50 value to inhibit Aβ-induced hippocampal neuron death is ~ 10 nM) [96]. LM11A-31 also crossed the blood-spinal cord barrier after being orally administered to mice, reaching an effective concentration in plasma and spinal cord after injury [67]. Favorable mouse CNS uptake was suggested by brain-to-plasma ratios greater than one following both acute and chronic administration [93]. Therapeutic CNS levels have also been described in cats [97] following oral administration and in rats after intraperitoneal injection [98]. In all, these studies show that LM11A-31 is CNS permeable, reaching effective concentrations in target tissues.

Evaluating potential side effects, such as changes in body weight and pain sensitization, provides important information on the safety and tolerability of LM11A-31, and more generally on potential side effects of small molecules targeting p75^NTR^. Given that LM11A-31 promotes NGF-like signaling via p75^NTR^, and NGF-TrkA signaling is known to cause neuropathic pain [99, 100], evaluating its effects on nociception is essential for its therapeutic development. Three separate mouse studies have shown that LM11A-31 administration does not induce hyperalgesia relative to vehicle treatment in assays of heat sensitivity and mechanical allodynia [67, 93, 94], in contrast to NGF, which triggers heat-induced hyperalgesia [93]. Neurotrophic factors have been linked to body weight regulation; for example, disrupting BDNF signaling can reduce satiety, leading to obesity [101]. Thus, effects of LM11A-31 on body weight required investigation. LM11A-31 did not affect body weight when administered orally at doses ranging from 5 to 100 mg/kg (once daily treatment with a duration range of 7 days to 1.5 to 3 months) to aged mice [102]; mouse models (and wild-type controls) of AD [93], HD [103], diabetic retinopathy [81], and skeletal muscle [104] and spinal cord injuries [67]; as well as a feline HIV model [97] and a rat model of bacterial meningitis [70]. At higher doses, it produced a non-significant reduction in body weight in mice with peripheral nerve injury after 17 days of daily oral treatment with 50, 100, and 200 mg/kg [68]. Furthermore, no toxicity was observed after single escalating doses of LM11A-31 up to 2000 mg/kg [67]. Thus, the safety and tolerability of LM11A-31 have been established in multiple species, preclinical pathological models, and treatment paradigms. Together, these initial studies showed that LM11A-31 exerts effects via p75^NTR^ and is a brain-penetrant, water-soluble, and well-tolerated small molecule that can modulate p75^NTR^ signaling to promote trophic signaling, inhibit degenerative signaling, and antagonize proNGF binding to p75^NTR^.

### Preclinical applications of LM11A-31

LM11A-31 was initially characterized in the context of nervous system cell populations, including central and peripheral neurons and oligodendrocytes [90]. However, given the fundamental nature of p75^NTR^ function and its widespread expression by multiple cell types both within and outside of the nervous system, as well as the favorable drug properties of LM11A-31, it has been applied by many laboratories across a wide range of pre-clinical models. Given this biological range, we sought to review all published applications of LM11A-31 in order to derive a broad overview of its effects which may be utilized to create a novel approach for further capturing the biology of p75^NTR^. In addition, this review points to potential clinical applications of LM11A-31 and profiles the potential for its therapeutic use.

We queried PubMed and Google with the search term “LM11A-31” and found 63 peer-reviewed, published studies utilizing LM11A-31 (as of March 10, 2026): 62 preclinical studies, including the initial publication reporting its discovery [90], and one human clinical trial [105]. The clinical trial is discussed at the end of this review, and the preclinical studies of LM11A-31 are summarized in Tables 1 and 2. Table 1 comprises preclinical studies investigating the therapeutic efficacy of LM11A-31. Table 2 includes studies using LM11A-31 to primarily address mechanistic questions regarding the role of p75^NTR^ in a particular pathological setting [138–142]. Both tables include the pathological condition and cellular/animal model investigated, the intended effect on p75^NTR^ (including modulation, antagonism, inhibition), route of administration, effective dose and dose duration, and LM11A-31’s statistically significant effects on outcomes related to that particular pathological condition. For the effective dose, a dose was included if it was effective against at least one endpoint in the study. We also noted whether the study was one of our laboratories’ publications (Longo and/or Massa), collaborative between our laboratories and another group, or conducted by a research group independent of our laboratories.

**Table 1:** Effects of targeting p75^NTR^ with LM11A-31 in preclinical studies of therapeutic efficacy. Fifty-six preclinical studies investigating the therapeutic efficacy of targeting p75^NTR^ with LM11A-31 were identified in our literature search and are summarized in this table. The pathological condition, cellular and/or animal model investigated, the effective dose, route of administration (ROA), and dose duration of LM11A-31, the p75^NTR^ mechanism targeted with LM11A-31, and LM11A-31’s effects on outcomes that were statistically significant are provided, unless they were noted as unchanged. Additionally, whether the study was conducted by the Longo and/or Massa laboratories, through a collaboration with the Longo and/or Massa laboratories, or by an independent research group was noted. For in vivo experiments, the sex of the animal is provided in parentheses in the animal model column. For the effective dose, a dose (or concentration for in vitro studies) was included if it was effective against at least one endpoint in the study; in vivo doses were delivered once daily unless otherwise noted

Pathological condition	Species – model (sex)	**Intended ** **effect on ** **p75**^**NTR**^	Effective dose, ROA, duration	LM11A-31 effects	Research group	Reference
Alzheimer’s disease	Mouse - cultured CF1 cortical, hippocampal, and septal neurons + Aβ; C57BL/6J hippocampal slices + Aβ; Rat - Wistar - hippocampal slices + Aβ	modulation	5–500 nM, CM	Prevented Aβ-induced AKT and CREB inactivation, calpain/CDK5, GSK3β, and c-Jun activation, and neurite dystrophy of hippocampal neurons; protected hippocampal, cortical, and septal neurons, and hippocampal slices from Aβ-induced cell death; prevented Aβ-induced LTP deficits in hippocampal slices	Longo Massa	Yang T PLoS One 2008 [96]
Alzheimer’s disease	Mouse - APP^L/S^ (f)	modulation	50 mg/kg, PO, 3 mo	Reduced degeneration of cholinergic neurites in basal forebrain and cortex; reduced neurite dystrophy in hippocampus; prevented cognitive deficits; did not reduce Aβ levels in cortex or hippocampus; did not cause hyperalgesia	Longo Massa	Knowles JK Neurobiol Aging 2013 [93]
Alzheimer’s disease	Mouse - APP^L/S^ (m)	modulation	25, 50, or 100 mg/kg, PO, 3 mo	Increased hippocampal p75^NTR^ cleavage; reduced cortical tau hyperphosphorylation and misfolding; reduced activated microglia in hippocampus and cortex and reactive astrocytes in cortex; mitigated degeneration of basal forebrain cholinergic neurites and cholinergic terminals in cortex; prevented cognitive deficits	Longo Massa	Nguyen TV J Alzheimers Dis 2014 [94]
Alzheimer’s disease	Mouse - APP^L/S^ (m), Tg2576 (f)	modulation	50 mg/kg (APP), 119 mg/kg (Tg2576), PO, 1 or 3 mo	Normalized p75^NTR^ levels in basal forebrain; prevented and reversed dystrophy of cholinergic neurites in basal forebrain and cortex	Longo Massa	Simmons DA PLoS One 2014 [61]
Alzheimer’s disease	Mouse - APP^L/S^ (m)	modulation	50 mg/kg, PO, 3 mo	Reduced elevated microglial activation in cortex and hippocampus; reduced TSPO-radiotracer binding, as detected with PET imaging	Longo Massa	James ML Theranostics 2017 [106]
Alzheimer’s disease	Mouse - APP^L/S^ (m); cultured CF1 hippocampal neurons + Aβ	modulation	50 mg/kg, PO, 3 mo; 100 nM, CM	Reduced Aβ-induced hyperphosphorylation, misfolding, oligomerization, and mis-sorting of tau, prevented elevated activation of Fyn kinase and accumulation of its targets (pTauY18, pNR2B), reduced elevated RhoA signaling and cofilin activation and prevented degeneration of neurites and dendritic spine loss in hippocampal neurons; prevented elevated JNK, cofilin, and caspase3/7 activation, reduced pNR2B levels, decreased tau oligomerization, aggregation, and cleavage, and prevented decreased dendritic spine density in hippocampus	Longo Massa	Yang T Sci Rep 2020 [107]
Tauopathy	Mouse - Tau P301S (PS19) (m)	modulation	50 mg/kg, PO, 3 mo	Reduced elevated activation of calpain, cdk5, JNK, and cofilin; decreased tau hyperphosphorylation, acetylation, misfolding, fragmentation, aggregates, paired helical filaments, and seeding in hippocampus; reduced tau hyper- phosphorylation in hippocampal synapses; reduced hippocampal microglial activation, dendritic spine loss, and neurite and synapse degeneration; did not prevent hippocampal volume loss or astrogliosis; prevented spatial learning and memory deficits; extended lifespan	Longo Massa	Yang T Acta Neuropathol Commun 2020 [108]
Tauopathy	Mouse - Tau P301S (PS19) (m); cultured C57BL/6J hippocampal neurons + recombinant oligomeric Tau (oTau) or natural oTau-containing hippocampal insoluble fractions obtained from untreated or LM11A-31-treated PS19 mice	modulation	50 mg/kg, PO, 3 mo; 100 nM, CM	Prevented elevations in RhoA and cofilin activation and decreases in PKC and LIMK activation, reduced tau hyperphosphorylation (AT8, pTau Thr217) and oligomerization, and prevented dendritic degeneration and spine loss (partially dependent on LIMK activity) in hippocampal neurons exposed to recombinant oTau or natural oTau from untreated PS19 mice; reduced pathological tau in hippocampal insoluble fractions from LM11A-31-treated PS19 mice; reduced pathological tau in hippocampal insoluble fractions from LM11A-31-treated PS19 mice and preserved dendritic integrity and spine density in hippocampal neurons exposed to these fractions	Longo Massa	Yang T Acta Neuropathol Commun 2026 [109]
Age-related neuro-degeneration	Mouse - C57BL/6J, aged (np)	modulation	50 mg/kg, PO, 1–4 mo	Reversed basal forebrain cholinergic neuron and neurite degeneration; reversed neurite degeneration and preserved cholinergic fiber target innervation in hippocampus and parietal cortex; reversed hippocampal and cortical synapse loss	Longo Massa	Xie Y Sci Rep 2019 [102]
Huntington’s disease	Mouse - R6/2 (m), BACHD (m)	modulation	50 mg/kg, PO, 2 mo (R6/2), 7 mo (BACHD)	Restored trophic (AKT) and inhibited degenerative (JNK) signaling, normalized NFκB, ROCK, PTEN signaling, normalized p75^NTR^ cleavage product levels in striatum; reduced huntingtin aggregates in striatum, cortex, and hippocampus; prevented striatal cholinergic interneuron neurite degeneration and synapse loss; alleviated hippocampal and striatal dendritic spine loss; decreased striatal and hippocampal microglial activation; ameliorated motor and cognitive deficits; extended lifespan	Longo Massa	Simmons DA Hum Mol Genet 2016 [103]
Huntington’s disease	Mouse - R6/2 (m), BACHD (m)	modulation	50 mg/kg, PO, 2 mo (R6/2), 7 mo (BACHD)	Reduced elevated microglial activation in striatum, cortex, and hippocampus; reduced TSPO-radiotracer binding, as detected with PET imaging	Longo	Simmons DA Hum Mol Genet 2018 [110]
Huntington’s disease	Mouse - R6/2 (m, f)	modulation	50 mg/kg, PO, 2 mo	Decreased elevated urinary p75^NTR^-ECD levels; reduced elevated plasma cytokine levels; normalized iron regulatory protein levels in striatum; preserved microstructural integrity and reduced striatal, pallidal, and cortical atrophy, as assessed with MRI	Longo	Simmons DA Neurotherapeutics 2021 [111]
Huntington’s disease	Mouse - R6/2 (m), zQ175dn (m); Human- cultured SH-SY5Y cells expressing mutant huntingtin (mHtt)	modulation	10, 25, 50, 100 mg/kg, PO, 2 mo q.d. (R6/2), 4 mo b.i.d. (zQ175); 200 nM, CM	Improved autophagic flux and reduced number of SH-SY5Y cells with mHtt aggregates; decreased mHtt aggregates and ameliorated autophagy and lysosomal pathway dysfunction in striatum	Longo	Simmons DA Neurotherapeutics 2025 [112]
Parkinson’s disease	Human – cultured LUHMES dopa- minergic neurons + oxidative stress (6-OHDA)	modulation	20 nM, CM	Inhibited JNK-dependent cleavage of p75^NTR^; protected dopaminergic neurons from cell death	Independent	Pokharel PV J Neurochem 2025 [113]
Parkinson’s disease	Human – cultured SH-SY5Y cells (neuroblastoma) exposed to rotenone	modulation	500 nM, CM	Reduced environmental toxin (rotenone)-induced α-synuclein aggregation, oxidative and nitrosative stress, and expression of NADPH oxidase regulatory subunits; normalized expression of Nrf2 (redox homeostasis regulator); enhanced protein S-glutathionylation; prevented downregulation of mitochondrial biogenesis transcription factors (PGC-1α, PPAR-α), preserved mitochondrial integrity; restored cholesterol homeostasis, normalized cholesterol regulatory proteins; prevented neurite degeneration, cell loss, and apoptosis	Independent	Pensabene D Neurochem Res 2025 [114]
Stroke - ischemic	Mouse - C57BL/6J with distal middle cerebral artery occlusion and hypoxia (m)	modulation	50 mg/kg, PO, 0.5 or 3 mo	Normalized changes in brain glucose metabolism and redox homeostasis, restored loss of neurotransmitter levels (serotonin, acetylcholine), reduced tau phosphorylation and microglial activation in white matter tracts of thalamus; did not alter chronic inflammatory response to stroke at the infarct site; reduced cortical atrophy and neuron loss; alleviated cholinergic neuron loss in medial septum; did not prevent the loss of cortical tyrosine hydroxylase containing fibers; reduced motor, cognitive, and psychological deficits	Collaboration	Nguyen TV J Pharmacol Exp Ther 2022 [115]
Stroke - ischemic	Mouse - C57BL/6J with middle cerebral artery occlusion (m); Mouse - cultured cortical astrocytes and endothelial cells with oxygen and glucose deprivation/reperfusion	inhibition	50 mg/kg, IV, single dose; 1000 nM, CM	Prevented elevated p65 activation (NFκB signaling) and upregulation of proteins that promote tight junction degradation (MMP-9, VEGF) in cultured astrocytes; prevented decreased tight junction protein (OCLN) expression in cultured endothelial cells; reduced cortical infarct size, brain edema, and BBB breakdown; ameliorated deficits in neurological function	Independent	Qin X Glia 2022 [116]
Stroke - ischemic	Mouse - NMRI with transient distal middle cerebral artery occlusion (m); Mouse - co-cultured primary cortical neurons and reactive astrocytes	modulation, antagonism (proNGF)	25 mg/kg, IP, 3 days or 0.5 mo b.i.d.; 80 nM, CM	Reduced astrocytic proNGF and cortical neuron caspase 3, JNK, and PARP1 activation, and improved cortical neuron survival in vitro; reduced microglial activation and cleaved caspase 3, normalized levels of p75^NTR^-ECD and -ICD in cortex; reduced infarct size and BBB leakage	Independent	Nasoohi S Exp Neurol 2023 [65]
Stroke - ischemic	Mouse - C57BL/6J with intraluminal suture-middle cerebral artery occlusion (m)	modulation	50 mg/kg, IP, single dose	Increased trophic (Akt) and decreased degenerative (JNK/BAX) signaling in brain but did not affect elevated RhoA or NFΚB signaling; reduced ischemic stroke complications (cerebral infarct size, edema, and hemorrhagic transformation)	Independent	Mirzahosseini G Mol Neurobiol 2024 [117]
Hypoxia - perinatal	Mouse - C57BL/6J with moderate perinatal hypoxia (m, f)	modulation	50 mg/kg, PO, 0.25 mo	Prevented decreased density of parvalbumin-containing cells and peri-somatic puncta in cortex; attenuated decreased cortical gamma activity and cognitive impairment in adulthood	Collaboration	Chattopadhyaya B Brain 2025 [118]
Hypoxia - intermittent	Mouse- C57BL/6J and cultured PC12 cells exposed to intermittent hypoxia (IH) (m)	modulation	1–6 mg/ml (200 ul/mouse), IV, single dose	Enhanced targeting of neuroprotective xenon-magnetic lipid bubbles (Xe-MLB) to IH-exposed PC12 cells; increased targeting of Xe-MLB to cortical and hippocampal neurons and enhanced the ability of Xe-MLB to reduce DNA damage (γ-H2AX) and cortical neuron death in IH mice	Independent	Sun Y J Control Release 2025 [119]
Retinopathy - diabetic	Mouse - C57BL/6J with streptozotocin-induced diabetes (m); Human - cultured retinal endothelial cells + proNGF	modulation	50 mg/kg, PO, 1 mo q2d; 200 nM, CM	Blocked proNGF-induced RhoA activation, occludin phosphorylation, tight junction protein redistribution, and barrier permeability in retinal endothelial cells; ameliorated elevated VEGF, TNF-α, and IL-1β levels and restored the proNGF/NGF ratio in retina; decreased serum and retinal TNF-α and IL-1β levels; reduced retinal micro-vasculature RhoA and Rac-1 activation and vascular permeability	Collaboration	Elshaer SL Diabetologia 2019 [81]
Retinopathy - ischemic	Mouse - C57BL6J/B6.129S4 + ischemia/reperfusion injury (np); cultured MSCs; Human - retinal endothelial cells + CM from LM11A-31-treated MSCs	modulation	50 mg/kg, PO, 3, 6, 9, or 12 days, q.o.d.; 200 nM, CM	Increased secretion of trophic and angiogenic factors (SDF-1α, VEGF-A, NGF) from MSCs, enhanced paracrine effects of MSCs on survival factor transcript expression and angiogenic response in human retinal endothelial cells; ameliorated visual acuity deficits	Collaboration	Elshaer SL Int J Mol Sci 2021 [120]
Traumatic brain injury	Rat - Sprague-Dawley with controlled cortical impact TBI (m); Rat - cultured hippocampal NPCs	modulation	500 ng/60 ul, IN, 0.5 mo; 0.1, 1, 10, 100 nM, CM	Decreased JNK signaling and promoted proliferation, survival, and differentiation of hippocampal NPCs in vitro; in cortex and/or hippocampus, promoted neuronal and NPC survival and neurogenesis and reduced gliogenesis and microglial activation; ameliorated learning deficits	Longo Massa	Shi J Stem Cells 2013 [64]
Traumatic brain injury	Rat - Sprague-Dawley with controlled cortical impact TBI (m)	modulation	50, 75 mg/kg, IP, 0.75 mo	Beneficial effects on a multi-dimensional outcome measure, including learning and memory and cell proliferation	Massa	Haefeli J Sci Rep 2017 [121]
Traumatic brain injury	Mouse - C57BL/6J with mild repetitive TBI (m)	modulation	50 mg/kg, IP, 7 days q.o.d.	Ameliorated axonal damage in the prefrontal cortex and cognitive deficits	Independent	Liu G Exp Neurol 2024 [122]
Spinal cord injury	Mouse - C57BL/6J with spinal cord injury (f)	antagonism (proNGF)	10, 25, 100 mg/kg, PO, 1.4 mo b.i.d.	Blocked proNGF binding to p75^NTR^, reduced JNK3 activation and cytochrome C levels, increased myelin sparing and oligodendrocyte survival in spinal cord; reduced lesion area and improved motor function recovery; did not exacerbate pain sensitivity	Collaboration	Tep C J Neurosci 2013 [67]
Spinal cord injury	Mouse - C57BL/6J with spinal cord injury (f)	antagonism (proNGF)	100 mg/kg, PO, 1 mo or IVT single dose	Ameliorated loss of glutamatergic inputs onto tyrosine-hydroxylase-positive neurons in the spinal cord; prevented morphological changes and apoptosis of umbrella cells in bladder, urothelial hyperplasia, and detrusor hypertrophy; ameliorated bladder dysfunction	Independent	Ryu JC J Clin Invest 2018 [123]
Spinal cord injury	Mouse - C57BL/6J with spinal cord injury (f); Human - cultured urothelium cell line (UROtsa) + proNGF	antagonism (proNGF)	100 mg/kg, PO, 3, 7, 10 or 14 days; 10 nM, CM	Blocked proNGF-induced JNK activation in cultured urothelial cells; prevented the loss of p75^NTR^ in bladder mucosa and detrusor; reduced elevated urinary p75^NTR^-ECD levels and activated mast cell numbers in bladder; ameliorated urothelial hyperplasia, barrier function loss, and bladder (voiding) dysfunction	Independent	Zaabbarova IV Neurourol Urodyn 2018 [95]
Spinal cord injury	Mouse - C57BL/6J with spinal cord injury (m)	antagonism (proNTs)	100 mg/kg, PO, 7 days	Alleviated hindlimb mobility deficits, bladder dysfunction, and white and gray matter degeneration in the spinal cord	Independent	Ikeda Y Continence (Amst) 2022 [124]
Chemotherapy-associated neurotoxicity	Rat - cultured hippocampal and cortical cells; co-cultured dorsal horn and dorsal root ganglion cells + taxol, cisplatin, and methotrexate	modulation	100 nM, CM	Attenuated increased RhoA activity and prevented dendrite degeneration in hippocampal neurons; reversed neurite degeneration in dorsal horn/dorsal root ganglion neurons	Collaboration	James SE Neurotoxicology 2008 [125]
Chemotherapy-induced peripheral neuropathy	Mouse - C57BL/6J with cisplatin-induced peripheral neuropathy (m, f); cultured cortical neurons + cisplatin	modulation	25, 50 mg/kg, IP, 2.5 mo; 100 nM, CM	Reduced elevated RhoA activity in cortical neurons and sural and sciatic nerves; reduced neuritic branching deficits in cortical neurons; alleviated reductions in myelination, fiber density, and abnormal nerve fiber morphology in sural and sciatic nerves; prevented deficits in touch perception	Collaboration	Friesland A Neurotoxicology 2014 [126]
Peripheral nerve injury	Rat - Sprague-Dawley with sciatic nerve resection + implanted nerve guidance conduits (np); Human - cultured adipose tissue-derived stem cells	modulation	2.5 MA group equivalents in hygrogel filler, 1.5 mo	Enhanced adhesion and proliferation of human adipose tissue-derived stem cells; increased nerve cable area and axonal density, and stimulated peripheral nerve regeneration in vivo	Independent	Kohn-Polster C Int J Mol Sci 2017 [127]
Peripheral nerve injury	Mouse - C57BL/6J with sciatic nerve transection (m, f)	antagonism	2.5 M in fibrin glue, local, 3 days	Increased motor axon regeneration and re-innervation of muscle fibers; enhanced functional recovery	Independent	McGregor C Eur J Neurosci 2021 [128]
Peripheral nerve injury	Mouse - C57BL/6J with cavernous nerve injury (m); cultured major pelvic ganglia + LPS	antagonism (proNGF)	50, 100, 200 mg/kg, PO, 17 days; 10 nM, CM	Normalized elevated p75^NTR^ in dorsal nerve bundle; normalized JNK and PI3K/AKT signaling in penile tissue; increased cavernous eNOS activation and mitigated loss of neurovascular content (cavernous endothelial cells, pericytes, and neuronal processes); mitigated erectile dysfunction	Collaboration	Yin GN J Sex Med 2021 [68]
Skeletal muscle injury	Mouse - C57BL/6J with sciatic nerve transection (m,f)	antagonism (proBDNF)	75 mg/kg, PO, 7 days	Reduced JNK activation and IL-6 and TGFβ levels in sciatic-denervated skeletal muscle	Independent	Aby K Life Sci 2021 [69]
Skeletal muscle injury	Mouse - C57BL/6J with hindlimb ischemia-reperfusion injury (m,f)	antagonism (proBDNF)	50 mg/kg, PO, 1, 3, or 7 days	Reduced proinflammatory macrophages in skeletal muscle	Independent	Aby K Biology 2023 [104]
Skeletal muscle injury	Mouse - C57BL/6J with hindlimb ischemia-reperfusion injury +/- LPS (m,f)	modulation, antagonism (proBDNF)	50 mg/kg, PO, 15 days	Reduced elevated JNK and NFκB signaling, decreased pro-inflammatory cytokine IL-1β while increasing anti-inflammatory IL-10 transcript levels in skeletal muscle, and decreased LPS-induced IL-6 levels in serum	Collaboration	Aby K Int J Mol Sci 2025 [7]
Arthritis	Human - juvenile idiopathic arthritis patient mononuclear cells from synovial fluid + LPS and proNGF	antagonism (proNGF)	10 nM, CM	Reduced elevated p38 and JNK signaling, and decreased IL-6 levels	Independent	Minnone G RMD Open 2017 [77]
Arthritis	Human - rheumatoid arthritis patient fibroblast-like synoviocytes + TNF-α, IL-1β, LPS, proNGF, or synovial fluid from patients with juvenile idiopathic arthritis	antagonism (proNGF)	10 nM, CM	Reduced proNGF-induced p38 and JNK activation and release of pro-inflammatory cytokines (IL-6, IL-8 and MCP1) from rheumatoid arthritis patient synovial fibroblasts	Independent	Farina L Front Immunol 2022 [76]
Interstitial cystitis/bladder pain syndrome	Mouse - C57BL/6J with cyclophosphamide-induced cystitis (f)	inhibition	100 mg/kg, IVT, 30 min infusion	Increased bladder capacity and decreased void frequency in acute cyclophosphamide-induced cystitis but decreased these measures in the chronic condition	Independent	Hsiang HW Front Urol 2022 [129]
Interstitial cystitis/bladder pain syndrome	Mouse - C57BL/6J with cyclophosphamide-induced cystitis (f)	inhibition	100 mg/kg, IVT, single dose	Reduced elevated ERK activation in urothelium and JNK activation in detrusor of the bladder	Independent	Hsiang HW Front Urol 2023 [78]
HIV	Cat - cultured forebrain neurons with FIV; feline neurons exposed to CM from FIV-feline macrophages (fMCM)	modulation	10 nM, CM	Stabilized fMCM-induced intracellular calcium in neurons and astrocytes; prevented FIV-induced morphology changes and neurite density loss in forebrain neurons; prevented increased microglial density; supported neuronal survival	Collaboration	Meeker RB J Neuroimmune Pharmacol 2012 [130]
HIV	Rat - cultured forebrain neurons with HIV gp120, LPS + IFNγ, or CM from gp120-treated human monocyte-derived macrophages	modulation	2, 10 nM, CM	Prevented reductions in Akt and CREB activation and mitochondrial transport, stabilized intracellular calcium levels, and ameliorated neurite loss and soma morphological changes of hippocampal/cortical neurons; prevented HIV- and LPS + IFNγ-induced neuronal death	Collaboration	Meeker RB Exp Neurol 2016 [131]
HIV	Mouse - HIV gp120 (m,f)	modulation	50 mg/kg, PO, 3–4 mo	Reduced microglia, but not astrocyte, density, prevented loss of tau-containing dendrite clusters, prevented dendrite dystrophy and density loss, and ameliorated the loss of parvalbumin-containing GABAergic cells in hippocampus	Collaboration	Xie Y Exp Neurol 2021 [72]
HIV	Cat - infected with FIV (m)	modulation	50 mg/kg, PO, 2, 18 mo b.i.d.	Reduced viral titers in CSF but not systemically; increased the CD4:CD8 T cell ratio; prevented cognitive decline	Collaboration	Fogle JE J Neurovirol 2021 [97]
HIV	Human - monocyte derived macrophages (hMDM) and rodent microglia with HIV gp120; Rat/Mouse-forebrain neurons + CM from hMDMs, rodent microglia, or exposed to HIV gp120	modulation	10 nM, CM	Prevented HIV-induced suppression of calcium spike frequency and neurotoxin release from macrophages; normalized the HIV-induced secretory profile of macrophages and microglia; promoted a less neurotoxic morphological phenotype of macrophages and suppressed their activation	Collaboration	Killebrew DA J Neuro- immune Pharmacol 2022 [132]
HIV	Human - monocyte-derived macrophages with HIV-1; HIV-1-uninfected U937 monocytic cell line; HIV-1-infected U1 monocytic cell line	modulation	100 nM, CM	Suppressed HIV-1 replication, reduced cytotoxicity, oxidative stress, and inflammatory cytokines in macrophages; no effect on autophagy or Akt activation	Independent	Mirzahosseini G Exp Biol Med Neurobiol 2024 [133]
Bacterial meningitis	Rat - Sprague-Dawley with Streptococcus pneumoniae (np)	modulation	15 ug/ 60 ul, IN, 3 or 7 days	Reduced elevated levels of proinflammatory transcription factors (NFκB/p65, C/EBPβ) and cytokines/mediators; decreased number of astrocytes and microglia in cortex and hippocampus; reduced IL-1β, TNF-α and iNOS in serum; reduced bacterial concentrations in spleen and cerebellum; reduced neuronal apoptosis and necrosis in hippocampus or cortex; increased hippocampal neurogenesis and neuron maturation; alleviated subarachnoid expansion and infiltration of inflammatory cells; ameliorated clinical severity scores; did not affect survival rate	Independent	Zhang D J Neuro-inflammation 2021 [70]
Sepsis-associated encephalopathy	Mouse - C57BL/6J with cecal ligation and puncture (m)	modulation	50 mg/kg, PO, 13 days	Prevented BDNF down-regulation and JNK activation; reduced elevated IBA1 and IL-1β but not TNF-α, IL-6, and IL-10; ameliorated dendritic spine loss and neuronal death in hippocampus; prevented cognitive deficits, did not extend lifespan	Independent	Ji M Behav Brain Res 2018 [83]
Cancer - ovarian	Human - cultured ovarian cancer cell lines (CAOV3, SKOV3, OVCAR3) + NGF	antagonism (NGF)	5 nM, CM	Prevented NGF-induced changes in expression of β-catenin and related genes and cancer cell migration	Independent	Li B Oncotarget 2016 [134]
Cancer - squamous cell carcinoma	Human - cultured cancer cell lines (PE/CA-PJ15, Cal-27, Detroit-562)	inhibition	15 nM, CM	Reduced cancer cell motility	Independent	Foerster Y Cells 2019 [135]
Epilepsy	Rat - Sprague-Dawley with pilocarpine-induced status epilepticus (np)	modulation	200 mg/kg, IP, two doses	Did not affect seizure severity, magnitude, progression of epileptogenesis or neuron death in the hippocampus	Collaboration	Grabenstatter HL J Neurosci Res 2014 [98]
Rett Syndrome	Human - fibroblasts derived from donors with Rett syndrome	modulation	100 nM, CM	Normalized expression of transcription factors involved in antioxidant and anti-inflammatory responses (PPARα, PPARγ) and redox homeostasis (PGC1α, Nrf2); prevented oxidative stress (nucleic acid damage and lipid peroxidation); partially restored dysregulated redox homeostasis; prevented increases in IL-6 and IL-8 in Rett fibroblasts	Independent	Varone M Biomedicines 2024 [84]
Alcohol use disorder	Rat - Long-Evans + alcohol (m)	antagonism (BDNF)	30 ug/ul, IC; 50, 100, 150 mg/kg, IP, single dose	Reduced binge-like alcohol intake via actions in the striatum	Collaboration	Darcq E J Neurosci 2016 [136]
Alcohol use disorder	Rat - Sprague- Dawley with adolescent intermittent ethanol exposure (m,f)	modulation	50 mg/kg, IG, 1 mo b.i.d q4d	Prevented loss of TrkA-containing cholinergic neurons in the nucleus basalis magnocellularis and deficits in acetylcholine activity in the medial prefrontal cortex and performance during a sustained attention task	Independent	Kipp BT Int J Mol Sci 2024 [137]

Abbreviations: b.i.d.: twice daily; CM: culture medium; f: female; FIV: feline immunodeficiency virus; HIV: human immunodeficiency virus; IC: intracerebral; IG: intragastric; IH: intermittent hypoxia; IN: intranasal; IP: intraperitoneal; IV: intravenous; IVT: intravesical (bladder); LTP: long-term potentiation; m: male; mo: month; MSCs: mesenchymal stem cells; np: not provided; NPCs: neural progenitor cells; NTs: neurotrophins; p: phosphorylated; PO: per os, including any route by mouth (e.g. oral gavage, drinking water, food); q.d.: once daily; q.o.d.: every other day; q2d: every 2 days; q4d: every 4 days; ROA: route of administration; SSH: slingshot phosphatase; TBI: traumatic brain injury

**Table 2:** Preclinical studies using LM11A-31 to address the role of p75^NTR^ in various pathological conditions. Six studies were identified that primarily used LM11A-31 to address mechanistic questions regarding the role of p75^NTR^ in a particular pathological setting. These studies, including the original paper reporting the discovery and initial characterization of LM11A-31, are summarized in this table. The pathological condition, cellular and/or animal model investigated, the effective dose, route of administration (ROA), and dose duration of LM11A-31, the p75^NTR^ mechanism targeted with LM11A-31, and LM11A-31’s effects on outcomes that were statistically significant are provided, unless they were noted as unchanged. Additionally, whether the study was conducted by the Longo and/or Massa laboratories, through a collaboration with the Longo and/or Massa laboratories, or by an independent research group was noted. For in vivo experiments, the sex of the animal is provided in parentheses in the animal model column

Pathological condition	Species – model (sex)	Intended effect on p75^NTR^	Effective dose, ROA, duration	LM11A-31 effects	Research group	Reference
Identify small molecule ligands for p75^NTR^ and profile their effects	Mouse - p75^NTR^+/+ and p75^NTR^−/− cultured embryonic hippocampal neurons; Rat - cultured cortical oligodendrocytes; NIH3T3 fibroblasts expressing null vector or p75^NTR^; 3T3–TrkA cells, PC12 cells	antagonism (proNGF), modulation	1, 5, 10 nM CM	Bound to p75^NTR^ and inhibited proNGF binding to p75^NTR^ in vitro; recruited IRAK to p75^NTR^ in PC12 cells; activated PI3K/Akt signaling in p75^NTR^-dependent manner, activated NFκB; inhibited apoptosis in hippocampal neurons; demonstrated that effects were mediated by p75^NTR^ and not by TrkA or TrkB activation; promoted survival of mouse hippocampal neurons; inhibited proNGF-induced oligodendrocyte death	Longo Massa	Massa SM J Neurosci 2006 [90]
Probe role of BDNF/p75^NTR^ in peripheral nerve injury	Spinal cord slice culture with BDNF treatment	antagonism (BDNF)	100 nM, CM	Increased frequency of spontaneous excitatory post-synaptic currents; did not alter BDNF-induced excitability	Collaboration	Boakye PA J Neurophysiol 2019 [138]
Probe role of p75^NTR^ ligands/p75^NTR^ in developmental dendrite and axon pruning; axon degeneration in neurodegenerative diseases	Mouse - C57BL/6J.129S - cultured sympathetic neurons with NGF withdrawal	antagonism (unspecified pro-degenerative ligand)	2 nM, CM	Inhibited p75^NTR^-dependent spheroid formation, which leads to axon degeneration	Independent	Yong Y J Neurosci 2019 [139]
Probe role of proNGF/p75^NTR^ in neurodegeneration and cancer	Cultured PC12 cells, serum starved	antagonism (proNGF)	10 mM, CM	Reduced cell death; blocked proNGF-A protective effects against cell death, suggesting that the ability of proNGF-A to prevent cell death involves p75^NTR^ activation; blocked the ability of NGF to increase the % of neurite bearing cells and neurite length indicating that these effects are partially p75^NTR^ dependent	Independent	Soligo M Neurochem Int 2019 [140]
Probe role of p75^NTR^ in antidepressant effects on post-traumatic stress disorder	Rat - Wistar + antidepressants (fluoxetine and ketamine) (m,f)	antagonism (anti-depressants)	1 mg/kg, IP, single dose; 1 µm, IC, single dose	Blocked antidepressant-induced increases in hippocampal LTP and improvements in extinction memory	Independent	Diniz C J Biopsych 2025 [141]
Probe role of proBDNF/p75^NTR^ in peripheral nerve injury	Mouse - C57BL/6J peroneal nerve with crush injury (np)	antagonism (proBDNF)	10 µm, bath application	No direct effect on synaptic transmission in regenerating neuromuscular junctions (NMJs); did not prevent proBDNF effects on synaptic transmission in regenerating NMJs indicating that proBDNF exerts these effects via a p75^NTR^-independent pathway	Independent	Bogacheva PO Neurochem Res 2025 [142]

Abbreviations: CM: culture medium; f: female; IC: intracerebral; IP: intraperitoneal; LTP: long-term potentiation; m: male; np: not provided; oTau: oligomeric Tau; ROA: route of administration

Since the initial work identifying LM11A-31 as a p75^NTR^ modulator [90], LM11A-31 has been tested in preclinical studies using cellular and animal models of 26 distinct disease and injury conditions (Table 3), reflecting its broad therapeutic potential. These studies include models of neurodegenerative diseases such as AD, HD, and PD; conditions related to hypoxia, injury/toxicity, and inflammation/infection, as well as cancer, Rett syndrome, epilepsy, and alcohol use disorder (Tables 1, 3). Six studies, including the original paper reporting the discovery and initial characterization of LM11A-31, addressed primarily mechanistic questions regarding the role of p75^NTR^ and its ligands in cell survival, cancer, developmental dendrite and axon pruning, peripheral nerve injury, and post-traumatic stress disorder (Table 2). Our research groups (Longo and/or Massa) contributed 16 of the 62 preclinical studies (26%) investigating the efficacy of LM11A-31 in multiple cellular and mouse models of AD, HD, tauopathy, and TBI (Table 1). Collaborative work, mainly or solely conducted in other laboratories, evaluated LM11A-31’s effects against pathology in 27% (17 of 62) preclinical studies of various conditions, including alcohol use disorder, perinatal hypoxia, HIV, and stroke. Further, 47% (29 of 62) of the preclinical and mechanistic studies of LM11A-31 were conducted by independent research groups across 22 different laboratories (Tables 1, 2). In some instances, multiple independent research groups showed beneficial effects of LM11A-31 for a particular condition. For example, three groups tested LM11A-31 against pathologies related to ischemic stroke and three others for peripheral nerve injury, while two different research groups each evaluated LM11A-31 in the context of TBI, spinal cord injury, HIV, and alcohol use disorder (Table 1). These repeated independent findings with LM11A-31 demonstrate the robustness and reproducibility of LM11A-31 outcomes and highlight p75^NTR^’s broad biological and pathophysiological significance.

**Table 3:** LM11A-31 preclinical therapeutic studies: applications and categories of pathological conditions. The left column indicates the specific pathological condition for which LM11A-31 was applied, the middle column shows the number of publications for each application, and the right column groups applications into broad categories of pathological contexts

Application	Number of publications	Category
Alzheimer’s disease	6	Neurodegeneration
Huntington’s disease	4	Neurodegeneration
Parkinson’s disease	2	Neurodegeneration
Tauopathy	2	Neurodegeneration
Age-related neurodegeneration	1	Neurodegeneration
Stroke - ischemic	4	Hypoxia
Hypoxia - perinatal	1	Hypoxia
Hypoxia - intermittent	1	Hypoxia
Retinopathy - ischemic	1	Hypoxia
Retinopathy - diabetic	1	Hypoxia
Traumatic brain injury	3	Injury/Toxicity
Spinal cord injury	4	Injury/Toxicity
Chemotherapy-associated neurotoxicity	1	Injury/Toxicity
Chemotherapy-induced peripheral neuropathy	1	Injury/Toxicity
Peripheral nerve injury	3	Injury/Toxicity
Skeletal muscle injury	3	Injury/Toxicity
Arthritis	2	Inflammation/Infection
Interstitial cystitis/bladder pain syndrome	2	Inflammation/Infection
HIV	6	Inflammation/Infection
Bacterial meningitis	1	Inflammation/Infection
Sepsis-associated encephalopathy	1	Inflammation/Infection
Cancer - squamous cell carcinoma	1	Cancer
Cancer - ovarian	1	Cancer
Rett Syndrome	1	Other
Epilepsy	1	Other
Alcohol use disorder	2	Other

### p75^NTR^ mechanisms targeted by LM11A-31 in preclinical applications

In preclinical studies evaluating the disease-modifying effects of LM11A-31, two mechanistic paradigms were employed: (1) modulation of p75^NTR^ with LM11A-31 to suppress degenerative signaling and promote trophic signaling, and (2) antagonizing ligand binding, primarily proneurotrophins, to p75^NTR^. In some studies, LM11A-31 was employed as a p75^NTR^ inhibitor with the general intent to inhibit degenerative signaling. In practice, LM11A-31 engages each of these mechanisms regardless of the mechanism targeted. Thus, LM11A-31’s intended uses described here denote the disease biology emphasized by the study authors rather than discrete drug actions (Table 1).

Modulating p75^NTR^ signaling with LM11A-31 was the therapeutic strategy consistently applied across neurodegenerative conditions, including AD and other tauopathies, HD, PD, and aging, as pathologies associated with these conditions trigger degenerative signaling cascades that highly overlap with p75^NTR^ signaling pathways and could therefore be offset by LM11A-31 (Tables 1, 3; Figs. 1, 2). p75^NTR^ modulation with LM11A-31 was also the strategy used in studies of Rett syndrome [84], epilepsy [98], TBI [64, 121, 122], chemotherapy-associated neurotoxicity/peripheral neuropathy [125, 126], and most hypoxia-related conditions (ischemic stroke, retinopathies, perinatal and intermittent hypoxia; see Table 1). Antagonism of proneurotrophin-p75^NTR^ binding was the approach exclusively applied in spinal cord injury studies to counteract elevated proneurotrophin (primarily proNGF) production by denervated tissues in the spinal cord and bladder wall [95, 123, 124]. Antagonism of proBDNF-p75^NTR^ binding was the basis for skeletal muscle injury studies [7, 69, 104]. Peripheral nerve injury studies were split between modulation and antagonism of neurotrophin or proneurotrophin binding to p75^NTR^ with LM11A-31 [68, 127, 128]. Regarding inflammatory conditions, blocking NGF/p75^NTR^ signaling or proNGF/p75^NTR^ binding using LM11A-31 was the strategy for alleviation of inflammation associated with bladder cystitis [78, 129] and arthritis [76, 77], respectively. LM11A-31 modulation of p75^NTR^ was the approach most utilized in infection models (HIV dementia, bacterial meningitis, sepsis-associated encephalopathy) to dampen inflammation, promote neuroprotection, and, in the case of HIV, to compensate for loss of neurotrophic support [70, 72, 83, 97, 130–133]. Blocking NGF binding to p75^NTR^ with LM11A-31 was the strategy employed in a cancer study, as both ligand and receptor are elevated with ovarian cancer [134, 135]. Finally, antagonizing BDNF/p75^NTR^ binding or modulating p75^NTR^ signaling with LM11A-31 were the intended approaches in studies of alcohol use disorder [136, 137]. Collectively, these studies illustrate the diverse rationales for applying LM11A-31 to address distinct disease mechanisms.

### LM11A-31 engages p75^NTR^ and triggers its associated intracellular signaling pathways

p75^NTR^ undergoes proteolysis upon ligand binding, and LM11A-31 regulates this process. Our laboratories demonstrated that when p75^NTR^ proteolysis was unchanged or reduced—such as in the hippocampus of AD and striatum of HD mouse models—LM11A-31 increased levels of its proteolytic products, the C-terminal fragment (CTF) and the ICD, relative to full-length p75^NTR^, consistent with in vivo engagement of the receptor [94, 103]. Other research groups found that LM11A-31 can block pathologically elevated p75^NTR^ proteolysis, normalizing elevated CTF and ICD levels in an oxidative stress-induced cellular model of PD [143] and normalizing increased levels of ECD and ICD in mouse brains after ischemic stroke [65]. These data support in vivo target engagement of p75^NTR^ by LM11A-31.

LM11A-31 also decreases levels of p75^NTR^ in various cell types and pathological conditions. This reduction may help restore the disrupted p75^NTR^ to Trk- or neurotrophin ratios that occur in these disease/injury states. LM11A-31 reduced levels of full-length p75^NTR^ in dorsal nerve bundles after peripheral nerve injury [68], in basal forebrain cholinergic neurons in an amyloid AD mouse model [61], in the striatum of HD mice [103], and in the cortex and hippocampus of mice with bacterial meningitis [70]. Conversely, no reduction in p75^NTR^ induction was seen with LM11A-31 treatment in the hippocampus following TBI in rats [64, 121, 122]. LM11A-31 prevented decreases in hippocampal BDNF expression in mice with sepsis, normalizing the proBDNF/p75NTR imbalance that occurs in this condition [83]. Finally, it also normalized the elevated proNGF/NGF ratio that occurs in the retinas of a mouse model of diabetes [81].

LM11A-31 modulates each of the main p75^NTR^ downstream signaling pathways, including JNK/c-Jun, PI3K/Akt, NFκB, and RhoA/ROCK/cofilin. JNK pathway activation is primarily associated with pro-apoptotic/degenerative and inflammatory signaling, and it is elevated in numerous cell types and pathological conditions (Figs. 1 and 2). Sixteen studies showed that LM11A-31 reduces elevated JNK activation in a variety of disease and injury models (Table 1). Specifically, LM11A-31 prevented elevated JNK activation in the striatum of two mouse models of HD [103], the hippocampus of tauopathy mice [108], Aβ-exposed hippocampal neurons [96], and reduced JNK activation in hippocampal neural progenitor cells [64]. LM11A-31 has also been shown to reduce elevated pro-apoptotic JNK/BAX signaling in the mouse brain following ischemic stroke [117] and JNK/PARP1 signaling in primary cortical neurons [65]. Further, it prevented elevated JNK activation in the hippocampus of mice with sepsis [83] and in penile tissue from mice with erectile dysfunction following peripheral nerve injury [68]. Relevant to spinal cord injury, LM11A-31 blocked proNGF-induced JNK activation in cultured human urothelial cells of the bladder [95] and reduced interactions of proNGF and p75^NTR^ in association with preventing increased JNK activation in injured spinal cords of mice [67]. LM11A-31 inhibited increased JNK activation in skeletal muscle after denervation [69] and ischemia-reperfusion injury [7], and in urothelial and detrusor tissue of the bladder in mice with cystitis [78]. In the context of inflammatory processes, LM11A-31 prevented elevated JNK activation in fibroblast-like synoviocytes and synovial fluid mononuclear cells from rheumatoid arthritis or juvenile idiopathic arthritis patients [76, 77].

In many cell types and injury states in which LM11A-31 prevented JNK activation, it also increased PI3K-Akt signaling, which promotes activation of a variety of neuroprotective/antiapoptotic pathways including those involving tumor protein p53 (p53) and B-cell lymphoma 2 (Bcl-2) family members, as well as inhibiting JNK activation via phosphorylation of Apoptosis Signal-regulated Kinase 1 (ASK1) [144]. LM11A-31 prevented decreased PI3K/Akt activation in the striatum of HD mice [103], Aβ-treated hippocampal neurons [96], in the mouse brain following ischemic stroke [117], and in mouse penile tissue after peripheral nerve injury [68]. It also prevented decreased Akt activation in rat hippocampal/cortical neurons treated with conditioned media from HIV-gp120-exposed human macrophages [131]. Thus, LM11A-31 may inhibit degenerative signaling and promote neuroprotective signaling in many diverse pathological conditions.

p75^NTR^ functions in neurite outgrowth and dendritic spine maintenance are mediated via the RhoA signaling pathway, which includes the downstream effectors ROCK, PTEN, and cofilin. RhoA GTPase overactivation occurs in many pathological states and is associated with reduced dendritic spine structural plasticity and density and inhibition of neurite growth by promoting actin cytoskeleton rigidity [37, 145, 146]. LM11A-31 reduced elevated RhoA activation that occurs in retinal microvasculature of diabetic mice [81] and in cultured neurons and peripheral nerves from mice exposed to chemotherapy drugs [125, 126]. In addition, it prevented elevated RhoA activation induced by Aβ [107] or oligomeric tau (oTau) in hippocampal neurons [109]. LM11A-31 also reduces downstream effectors of RhoA. It attenuated elevated ROCK and PTEN levels in the striatum of HD mice [103] and reduced activated cofilin in the hippocampus of tauopathy mice [108], changes accompanied by preserved dendritic spine density. The cofilin phosphatase slingshot (SSH) counteracts LIMK activity, and together they control the equilibrium between phosphorylated (inactive) and dephosphorylated (active) cofilin, which regulates dendritic spine density. LM11A-31 prevented oTau-induced decreases in LIMK activation and dendritic spine density, which was LIMK-dependent, in hippocampal neurons [109]. Thus, modulating LIMK and SSH signaling to favor cofilin phosphorylation (and thus inactivation), as seen with LM11A-31, is beneficial for maintaining dendritic spine density.

Modulating p75^NTR^ with LM11A-31 prevented elevated phosphorylation of the NFκB subunit p65 relative to its unphosphorylated form (phospho-p65/p65) in mouse skeletal muscle after ischemia-reperfusion injury [7], and upregulated IκB and blocked microglial activation in the striatum of HD mice [103]. It also reduced phospho-p65/p65 in astrocytes subjected to oxygen and glucose deprivation/reoxygenation in a cellular model of ischemic stroke [116]. Other signaling pathways affected by LM11A-31 include those involving p38 and Fyn kinases. LM11A-31 reduced elevated p38 signaling in synoviocytes and mononuclear cells from rheumatoid arthritis patients [77] and reduced elevated Fyn signaling, which is associated with tau mis-sorting and synaptotoxicity, in the hippocampus in an AD mouse model [107].

### LM11A-31 reduces tau pathology

Tau pathology is present in a variety of neurological conditions, most notably AD and related dementias, and is a prioritized therapeutic target. The tau protein is found in axons where it stabilizes microtubules, which support structural integrity and neurite outgrowth. Post-translational modifications to tau, including hyperphosphorylation, drive pathological tau misfolding and aggregation and also disrupt the physiological functions of tau [147, 148]. Many aspects of tau pathology are inhibited by LM11A-31, which accords with the high overlap between p75^NTR^ signaling pathways and those disrupted in AD and other tauopathies. Many pathways controlled by p75^NTR^ regulate tau kinases. For example, Akt inhibits the major tau kinase GSK3b, and RhoA signaling activates ROCK, another tau kinase. p75^NTR^ signaling also influences activation of CDK5, JNK, and p38, all of which can phosphorylate tau [26, 30, 38, 94, 107, 149–151]. In tauopathy mice, LM11A-31 reduced pathological tau phosphorylation [108, 109] at four phospho-sites and reduced K280 acetylation, aggregation of tau oligomers, and tau seeding in hippocampus [108]. It also prevented tau hyperphosphorylation in LM11A-31-treated hippocampal neurons exposed to recombinant oTau or natural oTau contained in hippocampal insoluble fractions from tauopathy mice [109]. In an AD mouse model, it also reduced hyperphosphorylation, misfolding, cleavage, and oligomerization of tau in hippocampus and/or cortex [94, 107]. In the gp120 HIV mouse model, LM11A-31 preserved the p75^NTR^-dependent formation of putatively protective tau-containing clusters that occur in the hippocampus with aging [72]. Finally, LM11A-31 reduced the elevated tau phosphorylation seen in white matter tracts of the thalamus after ischemic stroke in mice [115].

### LM11A-31 preserves synapses and dendritic spines

Dendritic spine degeneration and synaptic dysfunction are fundamental pathologies underlying neurodegenerative diseases and other pathological conditions involving cognitive decline. Developing therapeutics that promote synaptic resilience is a high priority. Targeting p75^NTR^ for this purpose may be particularly appropriate given its well-established roles in synaptic plasticity and postnatal spinogenesis. p75^NTR^ signaling negatively modulates spine formation and dendrite morphology by modulating RhoA-ROCK-cofilin and JNK signaling [54]. Genetic ablation of p75^NTR^ increases spine density, while overexpression reduces it [152], and a function-blocking p75^NTR^ antibody prevented BDNF-induced increases in spine density and morphology in hippocampal slices [153]. Depending on the ligand and interaction partners, p75^NTR^ can promote opposing effects on synaptic plasticity. When activated by proBDNF, it enhances long-term depression, a process that weakens synaptic connections; when interacting with BDNF/TrkB, it enhances long-term potentiation (LTP), a process that strengthens synaptic connections [38].

Modulating p75^NTR^ with LM11A-31 promoted dendritic spine resilience and prevented synaptic dysfunction and loss associated with aging, sepsis-associated encephalopathy, and multiple neurodegenerative diseases. Our laboratories showed that LM11A-31 prevented and/or reversed dendritic spine loss in the hippocampus of an amyloid mouse model of AD [107] and a tauopathy mouse model [108], and in the striatum and hippocampus of two HD mouse models [103]. It also prevented the ~44–50% loss of dendritic spine density of hippocampal neurons exposed to oTau, restoring spine density to control levels [109]. LM11A-31’s positive regulation of spines was demonstrated by another research group, reporting that it almost completely prevented the ~52% reduction of hippocampal dendritic spine density in mice with sepsis [83]. In tauopathy mice, LM11A-31 also reduced levels of pathological tau in synapses and prevented the loss of pre- and post-synaptic proteins [108]. LM11A-31 ameliorated synapse loss in the hippocampus and cortex of aged mice [102] and prevented synaptic plasticity (LTP) deficits in hippocampal slices exposed to Aβ [96]. In each of these studies, the effect of LM11A-31 on dendritic spines was accompanied by normalization of spine-related signaling and recovery of cognitive and/or motor function.

### LM11A-31 prevents neurite and cell degeneration

Neuritic dystrophy and degeneration are common features of aging, age-related diseases, and brain injuries. They involve morphological changes, branching deficits, and reduced density of neuronal processes that often precede neuron loss and contribute to functional deficits, including cognitive and motor dysfunction. Pathologically elevated p75^NTR^ signaling occurring in various disease contexts may contribute to these changes by excessively activating RhoA/ROCK signaling to inhibit neurite outgrowth, activating apoptotic JNK and p38 signaling, and reducing pro-survival PI3K/Akt and NFκB signaling.

LM11A-31 prevented or reversed neurite dystrophy and neuronal death in vitro and in vivo in several different brain areas, including the hippocampus, cortex, striatum, and basal forebrain of mouse models of multiple pathological conditions. In cellular and animal models of HIV, LM11A-31 reduced the cytotoxicity of cultured HIV-infected macrophages [133], protected against dendrite damage and death of cortical/hippocampal neurons induced by FIV/HIV and macrophage-derived toxins [130, 131], and in the hippocampus of gp120 HIV transgenic mice prevented pathological changes in dendritic morphology and density loss, and ameliorated the loss of parvalbumin-containing GABAergic cells [72]. LM11A-31 reduced hippocampal neuron death in a mouse model of sepsis-associated encephalopathy [83], and, in a rat TBI model, stimulated hippocampal neural progenitor cell proliferation, enhanced neurogenesis and improved survival of hippocampal neurons, which was associated with improved learning [64]. LM11A-31 prevented neuritic dystrophy and death of mature hippocampal neurons exposed to Aβ [96], and reduced hippocampal neurite degeneration, particularly around amyloid plaques, in an AD mouse model [93]. It also promoted neurite complexity of hippocampal neurons exposed to oTau [109] and hippocampal pyramidal neurons in a tauopathy mouse model, although it did not prevent hippocampal volume reductions [108]. Finally, it partially reversed the loss of cholinergic innervation of the hippocampus in aged mice [102].

In the cerebral cortex, LM11A-31 improved neuron survival in mouse models of ischemic stroke [65, 115] and a rat model of TBI [64], and augmented the ability of xenon-magnetic lipid bubbles to reduce cortical neuron death in an intermittent hypoxia mouse model of obstructive sleep apnea [119]. LM11A-31 also alleviated axonal loss in the prefrontal cortex of mice with TBI [122]. In AD models, LM11A-31 improved survival of Aβ-exposed cultured cortical neurons [96] and reduced cortical clusters of dystrophic cholinergic neurites in AD mice [93, 94]. It also prevented the decrease in the density of parvalbumin-containing neurons and perisomatic puncta in cortex of mice with perinatal hypoxia [118]. LM11A-31 did not prevent the loss of tyrosine hydroxylase-containing fibers in the somatosensory cortex of stroked mice [115].

LM11A-31 mitigated cholinergic interneuron neurite dystrophy in the striatum of an HD mouse model, which was accompanied by normalized RhoA/ROCK/PTEN signaling [103], and prevented regional (striatal, cortical, and pallidal) and whole-brain atrophy in a mouse model of HD [111]. It also protected cells against neurite shortening and loss and cell death in in vitro PD models [113, 114].

Concordant with the high levels of p75^NTR^ in the basal forebrain, LM11A-31 prevented loss of cholinergic neurons in the nucleus basalis of Meynert of a rat model of alcohol use disorder [137] and in the medial septum in a mouse model of ischemic stroke [115]. It also reduced FIV-induced forebrain neuron and neurite degeneration [130] and protected cultured septal neurons from Aβ-induced cell death [96]. In an AD mouse model, LM11A-31 prevented cholinergic neurite dystrophy in the ventral diagonal band of the basal forebrain [93, 94]. Notably, when LM11A-31 was administered after pathology was manifest, it reversed cholinergic neurite dystrophy in the basal forebrain in AD mice [61] and reversed decreased basal forebrain cholinergic soma size and neurite length in aged mice [102]. The reversal of neurite and neuron degeneration by LM11A-31 suggests that modulation of p75^NTR^ combats pathology that has advanced beyond early stages, which, in the case of age-related diseases, may more accurately model the clinical population.

Spinal cord or peripheral nerve injury can induce elevated proneurotrophin and p75^NTR^ expression in motor neurons, oligodendrocytes, and Schwann cells, contributing to neurite degeneration and myelin deficits [58, 67, 154–156]. Several studies investigated the effects of LM11A-31 on axonal regeneration and remyelination in these conditions. LM11A-31 prevented white matter tract and grey matter degeneration throughout the spinal cord and improved hindlimb locomotion in mice with spinal cord injury [124]. The compound also prevented loss of glutamatergic inputs onto tyrosine-hydroxylase-positive neurons in dorsal root ganglia and L6/S1 spinal cords, and supported the survival of umbrella cells in the bladder [123]. LM11A-31 increased the number of myelinated axons and supported oligodendrocyte survival in the spinal cord after injury and improved motor coordination in these mice, consistent with its blocking proNGF binding to p75^NTR^ [67]. Regarding peripheral nerve injury, LM11A-31 promoted motor axon regeneration and their re-innervation of muscle fibers leading to enhanced functional recovery in mice with sciatic nerve transection [128]. After cavernous nerve injury, which can occur with prostatectomy, LM11A-31 enhanced cavernous endothelial cell, pericyte, and dorsal nerve bundle regeneration, ameliorating erectile dysfunction. This effect may partly reflect reduced inflammatory damage, as LM11A-31 also prevented LPS-induced neurite sprouting loss in ex vivo major pelvic ganglia [68]. Repair of severed peripheral nerves may be facilitated by bridges of artificial nerve guidance conduits that contain neuroprotective luminal fillers [127, 157]. Nerve guidance conduits with a filler containing LM11A-31 increased nerve cable area and axonal density when implanted into rats with sciatic nerve resection [127]. Peripheral neuropathy is a common complication induced by chemotherapeutic agents, including cisplatin and methotrexate, and has been associated with increased RhoA signaling [125, 158]. LM11A-31 decreased elevated RhoA activation and ameliorated the loss of neurite branching and/or length in cultured cortical neurons exposed to cisplatin [126] and cultured hippocampal or dorsal root ganglion neurons exposed to cisplatin or methotrexate [125]. It also alleviated reductions in hindpaw sural nerve myelination and the area and density of its fibers, and improved sensory function in mice with cisplatin-induced peripheral neuropathy [126].

In all, 19 studies showed that LM11A-31 protected neurites of varied neuronal types from degenerating. Neurites of basal forebrain cholinergic and hippocampal neurons were the most studied and were evaluated in a variety of pathological contexts. It also prevented the degeneration or death of multiple cell types, most prevalently neurons, in 21 studies using cellular or mouse models of neurodegenerative disease or injury (Table 1).

### LM11A-31 regulates inflammatory responses

Inflammatory responses are important drivers of pathology progression in a wide range of neurodegenerative diseases as well as deleterious CNS and systemic conditions. Inflammatory responses include glial cell activation and proliferation, and production and release of growth factors or chemo/cytokines. These responses can be regulated by p75^NTR^ via NFκB and JNK signaling. Twenty-three preclinical studies, including those from our laboratories and several independent groups, have consistently shown that LM11A-31 regulates inflammatory responses associated with cellular and animal models of 12 different pathological conditions, including neurodegenerative diseases (AD, HD, tauopathy), brain and spinal cord trauma and skeletal muscle injury, arthritis, HIV, bacterial meningitis, sepsis-associated encephalopathy, stroke, diabetic retinopathy, and Rett syndrome (Table 1).

LM11A-31 affects inflammatory responses associated with both microglia and astrocytes, as well as other immune cells. It reduced microglial activation and/or proliferation by about 20–75%, as measured by multiple microglial markers, including immunostained IBA-1 area, cell number and morphology, CD11b+ cells, and/or CD68 area in the cortex, hippocampus, and striatum of two HD mouse models [103, 110]; in the cortex and hippocampus of a rat TBI model [64], an amyloid mouse model of AD [94, 106], and a rat model of bacterial meningitis [70]; in the hippocampus of mouse models of tauopathy [108] and HIV gp120 [72]; and in the cortex [65] or thalamic white matter tracts [115] of two mouse models of ischemic stroke. LM11A-31 reduced the percentage of newly formed BrdU+/S100+ astrocytes following TBI by ~50%, bringing gliogenesis back to sham/vehicle levels [64]. Further, the compound ameliorated astrocyte reactivity or astrogliosis in the hippocampus and/or cortex in an amyloid mouse model of AD (~72% area of GFAP immunostaining) [94] and in a rat model of bacterial meningitis (~34–58% GFAP^+^ immunostained cells) [70]; but did not affect hippocampal GFAP levels in tauopathy mice [108] or number of GFAP-positive astrocytes in HIV gp120 mice [72]. Regarding other immune cells, LM11A-31 reduced the number of macrophages infiltrating injured muscle tissue [104] and reduced activated mast cell numbers in the bladder of mice following spinal cord injury [95]. It also decreased activation of HIV gp120-human monocyte-derived macrophages and produced morphological changes in macrophages consistent with decreased neurotoxicity [132], and normalized blood CD4:CD8 T-cell ratios in a feline HIV model [97].

Pro- and anti-inflammatory cytokines and chemokines are released from reactive microglia and astrocytes, and their sustained elevated levels can have deleterious effects, including eliciting cell death [159, 160]. We showed that LM11A-31 reduced elevated levels of multiple proinflammatory cytokines in the striatum [110] and plasma [111] of an HD mouse model. Other studies have also demonstrated that LM11A-31 lowers elevated proinflammatory cytokines in various disease models. LM11A-31 reduced inflammation-related transcription factors (NFκB/p65 and c/EBPβ) in rats with pneumococcal meningitis, which was accompanied by reduced cortical, hippocampal, and plasma levels of pro-inflammatory cytokines [70]. Among the various pro-inflammatory cytokines normalized by LM11A-31, interleukin (IL)-6 and IL-1β levels were consistently reduced across several studies. LM11A-31 also attenuated the release of cytokines from fibroblasts from patients with Rett syndrome [84] and decreased elevated pro-inflammatory cytokines induced by diabetes in retina and serum [81] and by sepsis-associated encephalopathy in the hippocampus [83]. LM11A-31 also changed the secretory profile of human monocyte-derived macrophages infected with HIV [132] and reduced their pro-inflammatory cytokine release [133]. In addition to preventing increased cytokine levels, LM11A-31 protected against cytokine-induced death of cultured macrophages in HIV models [131]. Multiple independent groups demonstrated that inhibiting inflammatory signaling with LM11A-31 reduced cytokine production or release in diverse cell and tissue types and pathological conditions. LM11A-31 attenuated the up-regulation of pro-inflammatory cytokines while increasing anti-inflammatory cytokines in skeletal muscle following sciatic denervation [69] and tourniquet-induced ischemia–reperfusion injury [7]. These changes were associated with reduced NFκB and JNK signaling in skeletal muscle following LM11A-31 treatment [7, 69]. LM11A-31 reduced JNK activation and pro-inflammatory cytokine release from synovial fluid fibroblasts and mononuclear cells from patients with juvenile idiopathic arthritis [76, 77]. In all, inflammation was the most prevalently studied and improved outcome of LM11A-31 treatment.

### Effects of LM11A-31 on functional outcomes

Modulating p75^NTR^ signaling with LM11A-31 and the resulting protective effects on disease- and injury-related pathologies often improved functional outcomes, including cognitive, motor, and psychological measures. LM11A-31 reduced cognitive deficits, primarily spatial, associative, and contextual learning and memory, in rodent models of AD [93, 94], HD [103], TBI [121, 122], HIV-associated neurocognitive disorder [97], tauopathy [108], stroke [115], sepsis-associated encephalopathy [83], and perinatal hypoxia [118]. LM11A-31 also prevented sustained attention deficits in a mouse model of alcohol use disorder [137]. LM11A-31 also improved cognition-related physiological measures including hippocampal LTP in slices treated with Aβ [96]; cortical gamma activity which is regulated by parvalbumin positive GABAergic interneurons during active exploration in a mouse model of perinatal hypoxia [118]; and prefrontal cortical cholinergic signaling during cue presentation and reward in a rat model of alcohol use disorder [137]. LM11A-31 also alleviated measures of psychological disturbances, including anxiety-like and compulsive behaviors that often accompany neurological disorders, in mouse models of AD [94], HD [103], and ischemic stroke [115].

Regarding motor dysfunction, LM11A-31 ameliorated deficits in balance, coordination, and gait in HD mice [103] and in mouse models of ischemic stroke, ameliorated deficits in motor [115] and sensorimotor function [65], as well as the neurological severity score, which includes several tests of motor ability [116]. In mice post-spinal cord injury, it alleviated hindlimb mobility deficits [124] and facilitated recovery of motor coordination [67]. LM11A-31 also enhanced recovery of compound muscle motor unit firing in mice after peripheral nerve injury [128]. Notably, LM11A-31 extended the survival of tauopathy mice by 33% [108] and of HD mice by 20% [103] with once daily treatment for 2-3 months. However, when delivered for shorter durations (3 to 13 days), LM11A-31 did not affect survival in rodent models of infection (e.g., sepsis and bacterial meningitis) [70, 83].

Other functional effects of LM11A-31 included: improving bladder function in mouse models of spinal cord injury [95, 123, 124]; increasing bladder capacity and decreasing void frequency in a mouse model of acute interstitial cystitis/bladder pain syndrome [129]; ameliorating a decline in visual acuity that occurs with ischemic retinopathy [120]; mitigating erectile dysfunction related to cavernous nerve injury [68]; preventing touch perception deficits associated with chemotherapy-induced peripheral neuropathy [126]; alleviating bacterial meningitis-induced decline in neurological scores [70]; and reducing binge-like alcohol intake in rats [136]. In contrast, LM11A-31 did not show therapeutic efficacy against epilepsy as it did not affect seizure severity, magnitude, or progression of epileptogenesis when administered to mice before and at the onset of status epilepticus [98]. Together, these results show that p75^NTR^ modulation can improve certain functional outcomes across a broad range of pathological conditions.

### Molecular translational endpoints reflecting LM11A-31 therapeutic effects

Translating potential therapeutics from preclinical studies to human clinical trials is greatly facilitated by non-invasive biomarkers relevant to both animals and patients. Effective biomarkers would detect a biological response relevant to a particular pathological condition, track disease/injury progression, and monitor therapeutic efficacy. Several preclinical studies have shown that LM11A-31 affects translational endpoints. Urinary p75^NTR^-ECD levels are increased with pro-apoptotic ligand binding and are excreted in urine and, as such, have been used as biomarkers in neurodegenerative diseases such as ALS [161]. LM11A-31 reduced levels of p75^NTR^-ECD in urine in an HD mouse model [111] and in mice with spinal cord injury [95]. Thus, urinary p75^NTR^-ECD may be a useful biomarker of pharmacological response and target engagement by LM11A-31. LM11A-31 suppressed the viral titer in CSF of a feline HIV model [97], and CSF viral load is used to monitor the efficacy of antiretroviral therapy in HIV patients [162]. Magnetic resonance imaging (MRI) is a translatable modality and has been used as a non-invasive way to detect structural changes in the brain and spinal cord of patients with numerous pathological conditions. Structural and diffusion MRI detected LM11A-31’s preventive effects on connectivity changes and regional and total brain volume loss in an HD mouse model [111]. Ex vivo MRI was also able to discern the sparing of white matter tracts in spinal cord seen with LM11A-31 treatment following injury in mice [124]. The reduction of microglial activation with LM11A-31 treatment was detected in vivo via positron emission tomography (PET) using radiotracers targeting translocator protein (TSPO) in one AD and two HD mouse models [106, 110]. TSPO-PET tracers have also detected neuroinflammation in AD and HD patients [163, 164], making it a potential molecular imaging biomarker of LM11A-31 treatment response, particularly in conditions involving neuroinflammation. Neuroinflammation also involves release of cytokines, which are elevated in the brains and plasma of patients and mouse models of neurodegenerative diseases [159, 165]. LM11A-31 reduced plasma levels of pro-inflammatory cytokines, including TNF-α and IL-1β, in an HD mouse model [111] and serum in a mouse model of diabetic retinopathy [81]. Together, these studies demonstrate that LM11A-31 alters several translational endpoints, including urine, plasma, CSF, and imaging markers relevant to CNS and inflammatory conditions. However, given the limited number of studies examining such endpoints following LM11A-31 administration, there is a clear need to further profile biomarkers of therapeutic efficacy as tools for translational and clinical development.

### Effects of LM11A-31 on emerging molecular and cellular functions of p75^NTR^

LM11A-31 also demonstrated effects on outcomes that were less well understood in terms of their relationship to p75^NTR^, potentially revealing novel insights into p75^NTR^ function. These included promoting autophagy and clearance of pathological protein aggregation, normalizing calcium and redox homeostasis, and reducing the breakdown of the blood-brain and blood-retinal barriers.

Autophagy is a process by which unnecessary, toxic, or damaged proteins are cleared from the cell. It is impaired in many neurodegenerative (e.g., AD, HD, and PD) and prion diseases, as well as systemic conditions like type 2 diabetes, contributing to a build-up of pathological protein aggregates that disturb normal cellular processes [166, 167]. Autophagy can be inhibited by p75^NTR^-mediated activation of the mammalian target of rapamycin (mTOR) via the PI3K/Akt pathway. p75^NTR^ can also promote autophagy via RhoA signaling to activate PTEN, a negative regulator of the PI3K pathway [168]. p75^NTR^ modulates mTOR levels and activation during aging [169] and in pathological conditions, including burn injury, cancer, and diabetes [170–172]. Independent of mTOR, p75^NTR^ can facilitate autophagy via interactions with TRAF6 and p62 (SQSTM1) [173]. These studies suggest that p75^NTR^ may bidirectionally regulate autophagy to affect the clearance of toxic protein accumulations that frequently occur in pathological conditions. Along these lines, modulating p75^NTR^ with LM11A-31 reduced the number of cells containing mutant huntingtin in a cellular HD model and decreased the number of intranuclear huntingtin aggregates in the striatum, hippocampus, and cortex of two different HD mouse models [103, 112]. This aggregate reduction was accompanied by molecular changes indicative of upregulated autophagy at multiple steps in the aggregate clearance process. LM11A-31 activated AMPK signaling and raised PTEN levels, both of which can initiate autophagy; boosted autophagic flux and normalized p62 (SQSTM1) levels, indicating enhanced autophagosome formation and cargo recognition; and finally, it increased mutant huntingtin association with lysosomes and elevated mature cathepsin D levels, which occurs during final degradation and clearance of protein aggregates [103, 112]. LM11A-31 also decreased α-synuclein accumulation in a cellular model of PD potentially by reducing oxidative stress which can contribute to protein misfolding and autophagy disruption [114]. In AD, misfolded Aβ and tau accumulate intra- and extracellularly and form insoluble amyloid plaques and neurofibrillary tangles, respectively. Interestingly, LM11A-31 reduced tau oligomers and insoluble tau without affecting amyloid deposition in an amyloid mouse model of AD [93, 94, 107], suggesting that it may interfere with Aβ-induced downstream degenerative signaling to mediate its effects on tau and other AD-related pathologies. Thus, p75^NTR^ modulation has the potential to promote the removal of deleterious protein aggregates in a host of pathological conditions and could do so by initiating autophagy. LM11A-31 will likely provide further important insights into the role of p75^NTR^ signaling in autophagy, and given the important role of autophagy in many human diseases, it may also provide a tractable therapeutic target for modulating this process.

Several lines of evidence suggest that p75^NTR^ plays a role in calcium homeostasis. In pituitary cells, NGF affects calcium currents via p75^NTR^ [174], and in neurons, proBDNF-p75^NTR^ signaling via the Rac1 pathway reduces calcium influx [175]. Modulating p75^NTR^ with LM11A-31 prevented the HIV-induced suppression of intrinsic calcium activity in macrophages, which robustly express the receptor, reducing their release of neurotoxic factors [132]. In cultured feline astrocytes and neurons, LM11A-31 prevented the late destabilization of intracellular calcium and reduced concomitant neuronal dendrite beading and pruning [130]. It also suppressed HIV (gp120)-treated macrophage-induced delayed calcium increases and activated Akt in cultured forebrain neurons, protecting them from damage [131, 132].

Redox homeostasis is disrupted in many pathological conditions, leading to oxidative stress, which can cause protein misfolding, mitochondrial dysfunction, and neuroinflammation [176]. Limited evidence suggests that p75^NTR^ signaling can influence redox homeostasis. Expression of p75^NTR^ or its ICD in PC12 cells protected against oxidative stress-induced cell death by maintaining glutathione in its reduced, active antioxidant form, indicating that p75^NTR^ exerts these effects independent of ligand binding [177]. p75^NTR^-deficient PC12 cells exhibited decreased mitochondrial membrane potential, elevated ROS, lowered glutathione synthesis, increased glutathione consumption, and impaired activation of the PI3K/Akt pathway under oxidative stress [178]. Under homeostatic conditions, p75^NTR^ signaling has been proposed to activate the transcription factor Nrf2, which promotes expression of antioxidant enzymes [179]. In a cellular PD model, modulating p75^NTR^ with LM11A-31 normalized elevated levels of Nrf2, reduced elevated expression of regulatory subunits of NADPH oxidase, which generates reactive oxygen species (ROS); enhanced protein S-glutathionylation, which protects against oxidative damage; normalized mitochondrial homeostasis, reducing ROS production; reduced ROS-associated cholesterol dysmetabolism; and decreased oxidative and nitrosative damage [114]. LM11A-31 also normalized chronic stroke-induced changes in glucose and redox homeostasis, including levels of reduced and oxidized glutathione [115], and reduced ROS in an HIV-1-infected monocytic/macrophage cell line [133]. In fibroblasts from Rett syndrome patients, modulating p75^NTR^ with LM11A-31 normalized expression of transcription factors involved in redox homeostasis, antioxidant, and anti-inflammatory responses, and partially restored dysregulated redox homeostasis by elevating reduced protein glutathionylation and lowering elevated Nox4 expression. In addition, it prevented oxidative stress, reflected by markers of nucleic acid damage and lipid peroxidation [84].

The BBB maintains CNS homeostasis by regulating the exchange of molecules and cells between the systemic circulation and the brain [180]. BBB selective permeability is mediated by its complex integrated structure of endothelial cells, pericytes, vascular smooth muscle cells, astrocytic endfeet, and basement membrane. BBB structural and functional integrity is compromised during aging, neurodegenerative disease (e.g., AD, PD), and other pathological conditions such as TBI and ischemic stroke [180–184]. The retina contains a blood-retinal barrier (BRB), with a structure and function similar to the BBB, that deteriorates with diabetic retinopathy [181–183, 185]. Interestingly, p75^NTR^ is present on several of the cell types regulating BBB and BRB function, including endothelial cells, vascular pericytes, and astrocytes, and is upregulated following insult or disease, including ischemic stroke [116, 186] and diabetic retinopathy [187]. This elevation in p75^NTR^ expression and signaling, as well as increased proNGF levels, has been associated with the degeneration of these cell types and BBB and BRB disruption [81, 116]. Concordantly, LM11A-31 improved BBB function in several studies evaluating mouse models of ischemic stroke by preventing BBB leakage when given immediately after stroke [65] and reduced BBB breakdown and associated brain edema and hemorrhagic transformation [116, 117]. LM11A-31 also improved BRB function by modulating RhoA activation to reduce proNGF-induced increases in the permeability of human retinal endothelial cells and retinal vasculature in a mouse model of diabetic retinopathy [81]. These functional improvements may be due to effects of LM11A-31 on tight-junction proteins which are a prime target of therapeutic agents aimed at stabilizing the BBB/BRB as they join endothelial cells to form the impermeable barrier that limits the entry of possibly harmful agents into the brain or retina [180, 188, 189]. Tight junctions are comprised of zonula occludens (ZO) and occludin proteins that are negatively regulated by vascular endothelial growth factor (VEGF) and matrix metalloproteinase-9 [180]. LM11A-31 reduced retinal VEGF expression, blocked proNGF-induced occludin phosphorylation (which degrades tight junctions), and prevented ZO-1 redistribution in diabetic mice with retinopathy [81]. In cellular models of ischemic stroke, LM11A-31 prevented astrocytic increases in matrix metalloproteinase-9 and VEGF levels and prevented occludin downregulation in endothelial cells subjected to oxygen and glucose deprivation/reoxygenation and treated with conditioned media from LM11A-31-treated astrocytes [116]. Given the pervasiveness of BBB disruption during disease and injury and the presence of p75^NTR^ on cells critical to barrier stability, modulating p75^NTR^ with LM11A-31 could preserve the BBB/BRB to provide neuroprotection during aging and many diverse pathological conditions.

### First-in-human clinical trial

While NGF has been administered in small-scale human trials [190, 191], the first human clinical trial evaluating selective targeting of p75^NTR^ was published in 2024, reporting the results of a phase 2a safety and exploratory endpoint trial of LM11A-31 in participants with mild to moderate AD dementia [105]. Characteristic of a phase 2a trial addressing a novel target, the primary outcome assessed was safety, which was successfully met. Secondary outcomes examined were CSF levels of core AD biomarkers including total tau, tau phosphorylated at Thr181 (p-tau181), Aβ40, and Aβ42. Although the study duration (26 weeks), small subject number, and resulting statistical power were insufficient to reliably determine effects on slowing cognitive decline, cognitive outcomes were incorporated as prespecified exploratory measures to evaluate directional trends, a procedure in line with typical phase 2a strategies in AD trials [192, 193]. Other prespecified exploratory outcomes included CSF levels of other biomarkers, structural MRI, and fluorodeoxyglucose (FDG)-PET. Significant drug-placebo differences were found for several secondary and exploratory endpoints, indicating that LM11A-31 slows progression of AD-related pathology. Specifically, LM11A-31 slowed or reversed longitudinal increases in CSF levels of biomarkers of synaptic degeneration, including synaptosomal-associated protein 25 (SNAP25) and neurogranin (NG), and the glial activation marker chitinase-3-like protein 1 (YKL40) [105], consistent with preclinical studies showing that LM11A-31 prevents synapse loss and reduces microglial and astrocytic activation in AD and tauopathy mouse models [107, 108]. In addition, longitudinal increases in CSF levels of Aβ42 and Aβ40 were slowed, though the ratio of Aβ42/Aβ40 remained unchanged. Total tau and p-tau181 levels were reduced but did not reach statistical significance. LM11A-31 also slowed gray matter loss (detected via quantitative structural MRI), and synaptic function decline (as indicated by glucose metabolism detected with FDG-PET imaging), in multiple cortical and hippocampal regions. While not statistically significant, LM11A-31 slowed the cognitive decline of participants by 50%, as measured by Mini-Mental State Examination and AD-assessment Scale-Cognitive Subscale, compared to placebo. Future trials on a larger scale with longer treatment duration will be needed to determine clinical efficacy [105]. Importantly, this study reinforces preclinical work related to LM11A-31’s effects on synaptic resilience and glial activation described above.

### Challenges of p75^NTR^ as a pharmacological target

Targeting p75^NTR^ with therapeutics such as LM11A-31 presents several potential biological, pharmacological, and translational challenges. Many of these challenges arise from attributes of the receptor, including broad expression across tissues, pleiotropic biological functions, and complex signaling that can promote survival, degenerative, death, and/or inflammatory signaling depending on the pathophysiologic context. Inhibition or excessive activation of p75^NTR^ can produce deleterious effects, such as upregulation of pain mechanisms or fibrosis, but this risk is mitigated by its very low or absent expression in many tissues under normal physiologic conditions and its upregulation only in response to injury and stress. Notably, LM11A-31 has shown no adverse effects in preclinical studies to date, and tolerability and safety endpoints were successfully met in the phase 2a clinical trial for LM11A-31 in participants with mild to moderate AD dementia [105]. In addition, small molecule approaches may be more prone to off-target effects and selectivity issues as p75^NTR^ shares structural similarities with other TNFRs, including a death domain-like signaling region and cysteine-rich extracellular domains. However, ligand cross-binding is unlikely since p75^NTR^ and TNFR ligand recognition sites are structurally distinct and their ligands (neurotrophins vs. cytokines [TNFα]) are structurally dissimilar [10]. Significant signaling crosstalk occurs between p75^NTR^ and other receptors, such as Trks, thus LM11A-31 may transactivate other p75^NTR^-dependent receptors. Finally, some p75^NTR^ signaling occurs independently of ligand binding, so therapeutics aimed at blocking ligand-receptor interactions may not fully inhibit degenerative signaling or deleterious functions. Though LM11A-31 appears to safely moderate p75^NTR^ activities, it will undergo continued careful study. Moreover, further efforts to target p75^NTR^ therapeutically should account for these potential issues and adopt appropriate controls or practices to manage or avoid them while harnessing the unique attributes of the receptor that make it a powerful target.

### Future directions

Given the breadth of disease models in which LM11A-31 has shown benefits, and its demonstrated safety and tolerability in humans, clinical trials for indications beyond AD may be imminent. Several disease areas are poised for clinical trials testing p75^NTR^ modulation, especially those characterized in numerous preclinical studies evaluating LM11A-31, including AD, HIV-associated neurocognitive disorders, peripheral nerve injury, HD, TBI, spinal cord injury, and stroke (Table 1). Other conditions in which p75^NTR^ contributes to pathology, for example, Down syndrome-related dementia [194], ALS [161], MS [74, 195, 196], and obesity/metabolic syndrome [197] represent additional opportunities for preclinical application of p75^NTR^ modulation. Testing LM11A-31 in such models could further define its therapeutic potential. In addition to its translational promise, LM11A-31 will continue to serve as a valuable tool to further elucidate p75^NTR^ functions in health and disease.

## Conclusions

Since its initial discovery and characterization, LM11A-31, the first-in-class small-molecule p75^NTR^ modulator, has been tested across 62 published mechanistic and preclinical studies in 26 distinct disease and injury applications. Across these studies, LM11A-31 normalizes changes in p75^NTR^ signaling, reduces inflammation, preserves synapses and dendritic spines, promotes neurite integrity, and prevents cell death that occurs in diverse diseases and injuries. Supporting the robustness of these effects, these outcomes have been replicated in multiple disease models and have been reproduced by multiple independent labs. A phase 2a clinical trial for AD confirmed safety and tolerability and showed biomarker and imaging changes consistent with preclinical findings, supporting its translational promise. Small-molecule modulation of p75^NTR^ appears to hold promise as a broadly useful therapeutic strategy for diseases that involve alterations in p75^NTR^ or neurotrophin signaling, or more generally, diseases that involve excessive inflammatory and degenerative signaling. Further studies are needed to define the full range of responsive cell types and promising disease applications. Clinically, next steps can include a larger, longer-duration trial of LM11A-31 in AD, as well as developing trials in areas that have been well studied preclinically, including AD-related disorders, tauopathies, HD, HIV-associated neurocognitive disorders, peripheral nerve injury, TBI, spinal cord injury, and stroke.

## Data Availability

Not applicable.

## Abbreviations

Aβ: Amyloid beta
AD: Alzheimer’s disease
ADAM17: A disintegrin and metalloprotease 17
Akt: Protein kinase B
ALS: Amyotrophic lateral sclerosis
BBB: Blood-brain barrier
BDNF: Brain-derived neurotrophic factor
BRB: Blood-retinal barrier
CNS: Central nervous system
CSF: Cerebrospinal fluid
ECD: Extracellular domain
ERK: Extracellular signal–regulated kinase
Fyn: A member of the Src family of tyrosine kinases
HD: Huntington’s disease
ICD: Intracellular domain
IKKβ: IκB kinase subunit beta
IκB: Inhibitor of NFκB
IL-1β: Interleukin-1 beta
IL-6: Interleukin-6
IRAK: Interleukin-1 receptor–associated kinase
JNK: c-Jun N-terminal kinase
LTP: Long-term potentiation
LIMK: LIM kinase
MAG: Myelin-associated glycoprotein
MAPK: Mitogen-activated protein kinase
mTOR: Mammalian target of rapamycin
NFκB: Nuclear factor kappa B
NG: Neurogranin
NGF: Nerve growth factor
NMDAR: N-methyl-D-aspartate receptor
NRAGE: Neurotrophin receptor-interacting melanoma antigen-encoding gene homologue
NT3: Neurotrophin 3
NT4/5: Neurotrophin 4/5
OMgp: Oligodendrocyte-myelin glycoprotein
p75^NTR^: p75 neurotrophin receptor
p75-ICD: p75 intracellular domain
p75-ECD: p75 extracellular domain
p-Tau181: Phosphorylated tau at threonine 181
PI3K: Phosphoinositide 3-kinase
PET: Positron emission tomography
PKC: Protein kinase C
PLCγ: Phospholipase C gamma
PNS: Peripheral nervous system
proNGF: Precursor nerve growth factor
proBDNF: Precursor brain-derived neurotrophic factor
PTEN: Phosphatase and tensin homologue
RhoA: Ras homolog family member A
ROCK: Rho-associated kinase
SNAP25: Synaptosomal-associated protein 25
SSH: Slingshot phosphatase
TACE: TNF-α converting enzyme
TBI: Traumatic brain injury
Trk: Tropomyosin receptor kinase
TrkA: Tropomyosin receptor kinase A
TrkB: Tropomyosin receptor kinase B
TrkC: Tropomyosin receptor kinase C
TSPO: Translocator protein
VEGF: Vascular endothelial growth factor
YKL40: Chitinase-3-like protein 1
